# BIRC3-CASP8 axis orchestrates the PANoptosis spectrum: taming the inflammatory storm to prevent post-ischemic heart failure

**DOI:** 10.3389/fimmu.2026.1875226

**Published:** 2026-06-29

**Authors:** Quancheng Han, Huajing Yuan, Yitao Xue, Yan Li, Yiding Yu

**Affiliations:** 1 First Clinical Medical College, Shandong University of Traditional Chinese Medicine, Jinan, China; 2 Department of Cardiology, Affiliated Hospital of Shandong University of Traditional Chinese Medicine, Jinan, China; 3 Department of Traditional Chinese Medicine, Shandong Provincial Hospital Affiliated to Shandong First Medical University, Jinan, China

**Keywords:** BIRC3, crosstalk, heart failure, PANoptosis, PANoptosome

## Abstract

**Background:**

The progression of heart failure (HF) following myocardial ischemia/reperfusion (I/R) injury is driven by regulated cell death. Unlike the restrained nature of apoptosis, pyroptosis and necroptosis are lytic processes that trigger inflammatory cascades, causing extensive collateral damage to the non-regenerative myocardium. Understanding the integrated regulation of these pathways (PANoptosis) is essential for limiting infarct expansion.

**Methods:**

We examined PANoptosis in rat I/R and H9c2 OGD/R models using transmission electron microscopy, immunofluorescence, and molecular markers (C-CASP3, N-GSDMD, p-MLKL). The functional hierarchy of the BIRC3-CASP8 axis was dissected using AAV-mediated gene transfer and pharmacological inhibitors.

**Results:**

We confirmed that I/R injury induces PANoptosis with interdependent crosstalk. Mechanistically, BIRC3 acted as a pivotal checkpoint: its upregulation inhibited CASP8, promoting membrane-rupturing pyroptosis and necroptosis. Crucially, BIRC3 silencing disinhibited CASP8, redirecting the cell death machinery toward apoptosis. This phenotypic shift preserved cell membrane integrity and minimized the release of inflammatory mediators, effectively halting the propagation of cell death to surrounding healthy cardiomyocytes.

**Conclusions:**

For cardiomyocytes destined to die, the BIRC3-CASP8 axis serves as a decisive switch between destructive and silent death modes. By leveraging this axis to shift PANoptosis toward an apoptosis-dominant phenotype, we can reduce the inflammatory storm and collateral injury. This offers a promising therapeutic paradigm to maximize the preservation of functional myocardium and arrest HF progression.

## 1 Introduction

Heart failure (HF) remains a leading cause of global morbidity and mortality, with myocardial ischemia/reperfusion (I/R) injury serving as a primary driver of its pathogenesis (1). Although timely reperfusion is critical for salvaging ischemic myocardium, restoration of blood flow can paradoxically exacerbate cardiomyocyte loss through oxidative stress, calcium overload, and inflammation (2). At the cellular level, distinct forms of programmed cell death (PCD), including apoptosis, pyroptosis, and necroptosis, have been implicated in this injury (3–5). However, these pathways have often been studied as separate processes, and the molecular crosstalk that integrates them during myocardial I/R injury remains incompletely understood.

Recent advances have defined PANoptosis as an inflammatory PCD modality that integrates molecular components of apoptosis, pyroptosis, and necroptosis (6). This process is mediated by the PANoptosome complex and may provide a framework for understanding why inhibition of individual death pathways does not always fully prevent cell death (7, 8). Although PANoptosis has been described in infectious and inflammatory diseases, its regulation and pathophysiological relevance in the non-regenerative myocardium during I/R injury remain largely unexplored (9).

The plasticity of PANoptosis may have important implications for myocardial injury. Pyroptosis and necroptosis are lytic forms of cell death associated with membrane rupture and the release of danger-associated molecular patterns and inflammatory cytokines, such as IL-1β, which may amplify local inflammation and contribute to collateral myocardial damage (10, 11). In contrast, apoptosis is generally considered a more restrained and immunologically silent process, characterized by preserved membrane integrity and orderly cellular clearance. Given the limited regenerative capacity of adult cardiomyocytes, modulating the mode of cell death may represent a potential strategy to reduce secondary injury after I/R.

**Figure 1:**
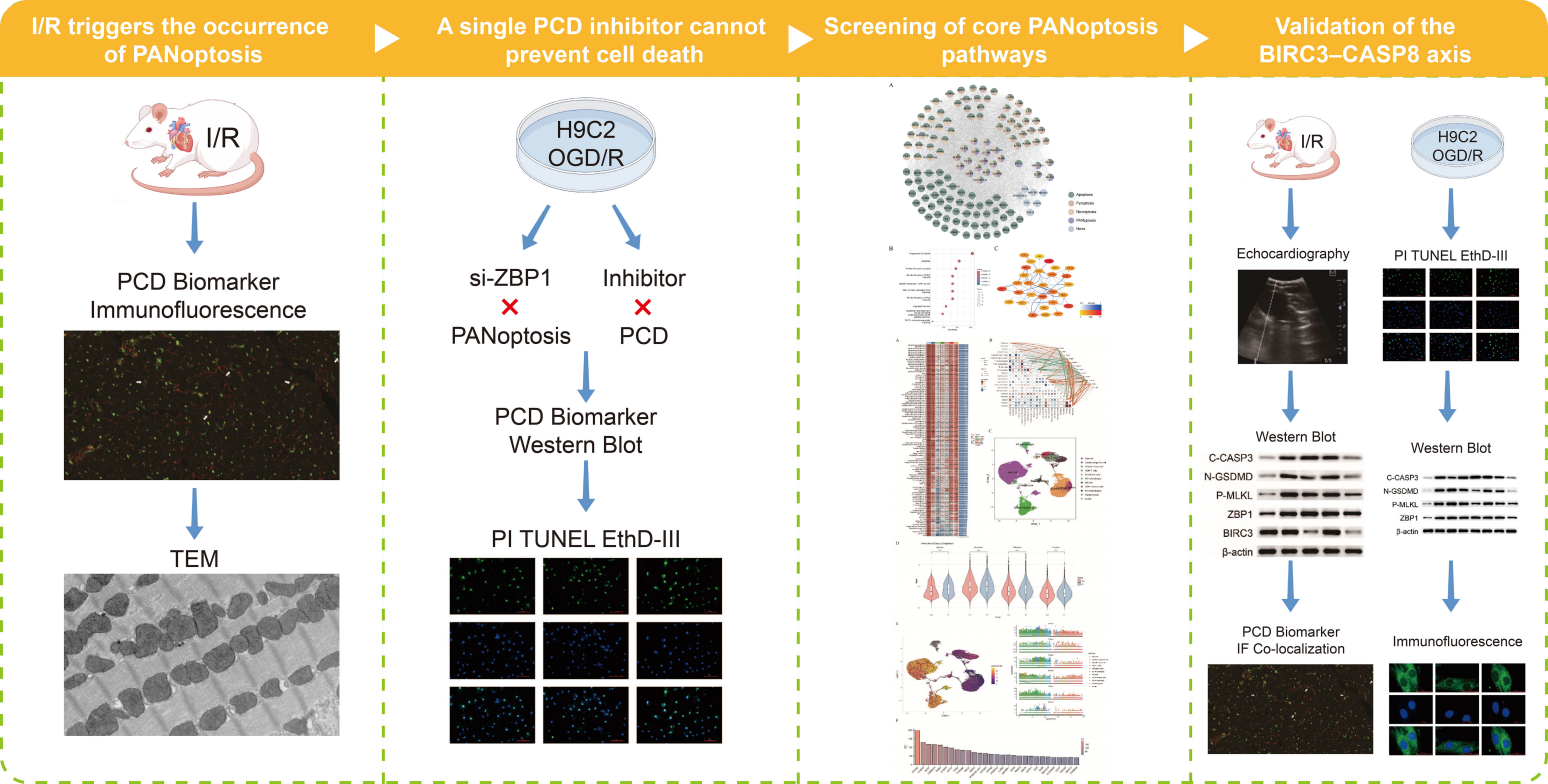
Study workflow. The workflow summarizes the experimental and analytical strategy used to investigate PANoptosis in myocardial ischemia-reperfusion injury, including validation of PANoptosis occurrence, assessment of single-pathway PCD inhibition, screening of core PANoptosis-related pathways, and experimental validation of the BIRC3-CASP8 axis. Abbreviations: I/R, ischemia/reperfusion; PANoptosis, an integrated programmed cell death process involving apoptosis, pyroptosis, and necroptosis; PCD, programmed cell death; TEM, transmission electron microscopy; H9c2, rat cardiomyoblast cell line H9c2; OGD/R, oxygen-glucose deprivation/reperfusion; si-ZBP1, small interfering RNA targeting Z-DNA binding protein 1; PI, propidium iodide; TUNEL, terminal deoxynucleotidyl transferase dUTP nick-end labeling; EthD-III, ethidium homodimer III; IF, immunofluorescence; BIRC3, baculoviral IAP repeat containing 3; CASP8, caspase-8.

In the present study, we aimed to investigate whether PANoptosis is activated during myocardial I/R injury and to explore the regulatory mechanisms underlying crosstalk among apoptosis, pyroptosis, and necroptosis. Using *in vivo* and *in vitro* models, together with bioinformatic and experimental approaches, we further examined the potential involvement of the BIRC3-CASP8 axis in regulating PANoptosis. The overall study workflow is shown in **Figure 1**.

## 2 Methods

### 2.1 Experimental animals and myocardial I/R model

All animal experiments were conducted in accordance with the Guidelines for the Care and Use of Laboratory Animals and were approved by the Institutional Animal Care and Use Committee of Affiliated Hospital of Shandong University of Traditional Chinese Medicine (Approval No. AWE-2023-010).

Male Wistar rats (age 6 weeks old, weighing 200 ± 20 g) were obtained from Beijing Vital River Laboratory Animal Technology (Laboratory Animal Quality Certificate Number: No.110011231106574714, License Number: SCXK (Beijing) 2021-0006) and housed under standard conditions (23 ± 2 °C, humidity 55 ± 5%, 12 h light/dark cycle, free access to food and water) (12).

After two weeks of adaptive feeding, to establish a myocardial ischemia/reperfusion (I/R) model, rats were anesthetized with an intraperitoneal injection of 4% sodium pentobarbital, their chest hair was removed, and they were intubated and mechanically ventilated (ventilator settings: 75 breaths/min, tidal volume 1.7 mL, 50:50 inspiratory/expiratory time ratio). An incision was made at the fourth and fifth intercostal spaces lateral to the left sternal margin. After layer-by-layer muscle separation, the chest cavity was opened, the pericardium was dissected, and the heart was exposed. A 6-0 3/8 circle arc suture needle was inserted 2 mm below the base of the left atrial appendage and exited adjacent to the pulmonary artery cone to ligate the left anterior descending artery. Successful ligation of the left anterior descending artery was confirmed by pallor at the apex, weakened cardiac activity, and ST-segment elevation on the electrocardiogram. Thirty minutes after ligation, the ligature was cut. The ECG showed a slight ST-segment decrease and a rosier apex, indicating restoration of perfusion. The chest was closed after layer-by-layer suturing. Postoperatively, the rats were kept warm, and penicillin was administered as needed to prevent infection. The sham surgery group only had suture threading performed. Similarly, the needle was inserted 2 mm below the base of the left atrial appendage and exited adjacent to the pulmonary artery cone. After 30 minutes of threading, the suture was removed, and the chest was closed with layer-by-layer suturing (13). Electrocardiograms and echocardiograms before and after ligation are provided in Supplementary File 1.

Rats in the treatment groups received Z-VAD-FMK (tail vein injection), AAV-mediated BIRC3 gene knockdown (intramyocardial injection), or a combination of these treatments.

Four weeks after modeling, cardiac function was first assessed using transthoracic echocardiography (Minray M5, Shenzhen Mindray Bio-Medical Electronics Co., Ltd.). M-mode images were obtained at the level of the papillary muscles, and left ventricular ejection fraction (LVEF) was calculated.

After echocardiographic evaluation, rats were deeply anesthetized with inhaled isoflurane (5% for induction and 3% for maintenance in oxygen). Adequate anesthesia was confirmed by the absence of the pedal withdrawal reflex and spontaneous movement. Subsequently, under deep anesthesia, rats were euthanized by exsanguination via abdominal aortic blood collection, and cardiac tissues were harvested for transmission electron microscopy, immunofluorescence, and Western blot analyses.

### 2.2 Transmission electron microscopy and immunofluorescence

Left ventricular myocardial tissues were rapidly excised and cut into small strips approximately 0.5 × 1 mm in size along the apical direction. The specimens were immediately immersed in 2.5% glutaraldehyde fixative solution (Solarbio, #P1127) and stored at 4 °C until transmission electron microscopy (TEM) examination. For TEM sample preparation, fixed myocardial tissues were rinsed three times with 0.1 M phosphate buffer (PB; 10 min each) to remove residual fixative, followed by post-fixation with 1% osmium tetroxide for 2 h to enhance contrast. After two additional washes with PB and two rinses with distilled water (10 min each), the samples were dehydrated through a graded ethanol series of 50%, 70%, 90%, and 100% ethanol for 15 min each, followed by two additional treatments with absolute ethanol for 10 min each. The tissues were then infiltrated sequentially with propylene oxide and Epon-812 resin mixtures at ratios of 1:1 and 1:4 for 1 h each, followed by infiltration with pure resin for 2 h. Subsequently, the samples were embedded in fresh resin, polymerized at 60 °C for 48 h, and sectioned using an ultramicrotome. Ultrathin sections were mounted on 200-mesh copper grids, double-stained with uranyl acetate and lead citrate, and examined using a transmission electron microscope (HT7800, Hitachi High-Technologies, Japan).

For immunofluorescence staining, rat myocardial tissues were fixed in 4% paraformaldehyde, dehydrated through a graded ethanol series, embedded in paraffin, and sectioned at a thickness of 5 μm. The sections were baked at 70 °C for 5 min, deparaffinized twice in xylene for 5 min each, and rehydrated through graded ethanol solutions of 100%, 95%, 85%, and 75% before being transferred to distilled water. Endogenous peroxidase activity was quenched with 3% hydrogen peroxide for 20 min at room temperature, followed by washing with PBS three times for 5 min each. After blocking with blocking solution for 30 min, the sections were incubated overnight at 4 °C with primary antibodies against cleaved caspase-3 (C-CASP3; CST, #9664), N-terminal gasdermin D (N-GSDMD; ABclonal, A24476), and phosphorylated MLKL (p-MLKL; ABclonal, AP1173). After washing, the sections were incubated with appropriate secondary antibodies for 40 min at room temperature in the dark. Fluorescent signal amplification was performed using 1× TSA dye working solution diluted 1:100, followed by antibody stripping at 37 °C for 20 min. The procedures of blocking, primary antibody incubation, secondary antibody incubation, TSA amplification, and antibody stripping were repeated sequentially for each target protein. Finally, nuclei were counterstained with DAPI for 10 min, and the sections were mounted with antifade mounting medium. Images were acquired using a digital slide scanner (Pannoramic MIDI II, 3DHISTECH, Hungary) and analyzed with a multispectral imaging system. The spatial localization and co-distribution of C-CASP3, N-GSDMD, and p-MLKL were assessed to determine whether these executor proteins overlapped in cardiomyocytes, thereby providing indirect evidence consistent with coordinated activation and colocalization of apoptotic, pyroptotic, and necroptotic executioner proteins.

### 2.3 Cell culture and OGD/R model

The rat cardiomyoblast cell line H9c2 was obtained from the Cell Bank of the Chinese Academy of Sciences (SCSP-5211, Shanghai, China). Prior to distribution, the cell line was confirmed to be free of mycoplasma, bacterial, and fungal contamination through quality control testing. Cells were cultured in high-glucose DMEM (L110KJ, Shanghai Yuanpei Biotechnology Co., Ltd., China) supplemented with 10% fetal bovine serum (FBS; S660JJ, Shanghai Yuanpei Biotechnology Co., Ltd., China) and 1% penicillin–streptomycin. The cells were maintained at 37 °C in a humidified incubator containing 95% air and 5% CO_2_. Cell dissociation was performed using trypsin solution (BL512A, Beijing Lanjieke Technology Co., Ltd., China).

To establish the oxygen–glucose deprivation/reperfusion (OGD/R) model, H9c2 cells were seeded into 24-well plates. Each well received 0.5 mL of complete medium, followed by the careful addition of 0.5 mL of cell suspension along the wall of the well to ensure uniform distribution. After gentle swirling, the plates were incubated for 24 h to allow cell attachment. Once the cells had adhered, one plate was designated as the control, or Sham, group, whereas the remaining plate was divided into model and treatment groups. After 24 h of pretreatment, the culture medium in all groups except the Sham group was replaced with glucose-free DMEM. The cells were then transferred to a hypoxic incubator containing 1% O_2_, 94% N_2_, and 5% CO_2_ for 6 h. Reperfusion was subsequently initiated by replacing the medium with fresh high-glucose DMEM and incubating the cells under normoxic conditions, consisting of 95% air and 5% CO_2_, for 4 h. The Sham group was maintained in complete medium under normoxic conditions throughout the experiment. After reperfusion, the cells were harvested for subsequent assays.

### 2.4 Inhibitor treatments

To evaluate the regulatory effects of programmed cell death inhibitors on OGD/R-induced PANoptosis in H9c2 cardiomyocytes, cells were treated with specific inhibitors targeting apoptosis, pyroptosis, and necroptosis. The apoptosis inhibitor Z-VAD-FMK (50 μM; MCE, HY-16658B), pyroptosis inhibitor disulfiram (0.3 μM; MCE, HY-B0240), and necroptosis inhibitor necrostatin-1 (Nec-1; 20 μM; MCE, HY-15760) were first dissolved in dimethyl sulfoxide (DMSO) to prepare stock solutions and then diluted in culture medium to the indicated final concentrations immediately before use. The final concentration of DMSO was kept identical among all groups, and an equivalent amount of DMSO was added to the vehicle control group.

### 2.5 Programmed cell death staining

To distinguish the three major forms of programmed cell death (PCD), TUNEL, propidium iodide (PI), and ethidium homodimer III (EthD-III) staining were performed. TUNEL staining detects DNA fragmentation by labeling free 3′-hydroxyl termini, thereby identifying apoptotic cells. Necroptosis is characterized by plasma membrane rupture, which permits PI entry into cells and subsequent nuclear staining. In contrast, during pyroptosis, N-terminal gasdermin D (N-GSDMD) inserts into the plasma membrane and forms membrane pores, increasing membrane permeability and allowing EthD-III to enter the cells and label nucleic acids (14). Complete membrane rupture during necroptosis may lead to nucleic acid release and incomplete fluorescence signals, thereby helping to distinguish necroptosis from pyroptosis.

TUNEL staining was performed to assess apoptotic cell death. The TUNEL working solution was freshly prepared by mixing 50 μL of TdT enzyme, 450 μL of fluorescein labeling solution, and 500 μL of TUNEL detection buffer. Cells were fixed with 4% paraformaldehyde for 30 min, permeabilized with 0.3% Triton X-100 for 5 min, and incubated with TUNEL working solution at 50 μL/well for 60 min at 37 °C in the dark. After washing, nuclei were counterstained with DAPI at 100 μL/well for 15 min.

PI staining was used to detect necroptotic cell death. A 500 nM PI working solution was prepared by diluting 1 mg/mL, or 1.5 mM, PI stock solution in 2× SSC buffer containing 0.3 M NaCl and 0.03 M sodium citrate at pH 7.0 at a ratio of 1:3000. Cells were fixed with methanol for 20 min, washed with 2× SSC buffer, permeabilized, and sequentially stained with DAPI at 100 μL/well for 5 min and PI at 300 μL/well for 5 min.

EthD-III staining was performed to identify pyroptotic cell death. Cells were fixed with 4% paraformaldehyde for 20 min, washed with PBS, and incubated with 3 μM EthD-III working solution at 200 μL/well for 20 min at room temperature. After washing, DAPI was added at 100 μL/well for nuclear counterstaining.

### 2.6 Immunofluorescence staining of cells

For immunofluorescence staining, the expression levels of the executor proteins cleaved caspase-3 (C-CASP3; apoptosis), N-terminal gasdermin D (N-GSDMD; pyroptosis), and phosphorylated MLKL (p-MLKL; necroptosis) in H9c2 cells were evaluated to assess PANoptosis activation. Briefly, cells were washed twice with PBS and fixed with 4% paraformaldehyde for 20 min at room temperature. After two washes with PBS for 2 min each, the cells were permeabilized with 0.1% Triton X-100 for 10 min, followed by another two PBS washes. The cells were then blocked with 5% BSA (A8020, Solarbio, Beijing, China) for 1 h at room temperature and washed with PBS. Subsequently, cells were incubated overnight at 4 °C with primary antibodies diluted in 1% BSA. After washing, the cells were incubated with fluorescently labeled secondary antibodies for 90 min at room temperature in the dark. Nuclei were counterstained with DAPI (C1005, Beyotime Biotechnology, Shanghai, China) for 10 min at room temperature. Finally, the cells were mounted using antifade mounting medium (S2100, Solarbio, Beijing, China), and fluorescence signals were captured using a fluorescence microscope.

### 2.7 Gene manipulation in H9c2 cells

To investigate the regulatory roles of BIRC3, CASP8, and ZBP1 in OGD/R-induced PANoptosis, gene overexpression and silencing experiments were performed in H9c2 cardiomyocytes.

Plasmid construction and overexpression: The full-length coding sequences of rat BIRC3 (NM_023987.3) and CASP8 (NM_022277.2) were cloned into the pcDNA3.1(+) expression vector (Invitrogen, USA). Plasmid DNA was purified using a midiprep kit (Beyotime, D0018) according to the manufacturer’s instructions. H9c2 cells were seeded into 6-well plates and transfected with 2.5 μg of plasmid DNA per well using Lipofectamine™ 3000 reagent (Invitrogen, L3000-008) in Opti-MEM medium (Invitrogen, 31985-070). After 6 h of transfection, the medium was replaced with complete DMEM supplemented with 10% FBS. Cells were cultured for 48 h before subsequent analyses.

siRNA design and transfection: To silence gene expression, small interfering RNAs (siRNAs) targeting BIRC3 and ZBP1 were synthesized by GenePharma (Shanghai, China). Three candidate siRNA sequences were designed for each gene as follows: si-BIRC3-1, 5′-GCAGCGACCUCAUUCAGAAAC-3′; si-BIRC3-2, 5′-CGUGUUAGAACGUUCUCUACC-3′; and si-BIRC3-3, 5′-AGAUGACAUUGCAGCUCUACC-3′. The siRNA sequences targeting ZBP1 were si-ZBP1-1, 5′-GGAAUGUUAUGUCAAGACAGA-3′; si-ZBP1-2, 5′-GCUGUUGAAGAAGUUACAAGU-3′; and si-ZBP1-3, 5′-GUUAUGUCAAGACAGACAAUC-3′. H9c2 cells were transfected with 50 nM siRNA using Lipofectamine™ 3000 reagent according to the manufacturer’s protocol. Cells were cultured for 48 h before subsequent analyses.

### 2.8 AAV-mediated BIRC3 knockdown in rats

Short hairpin RNA (shRNA) sequences targeting rat Birc3 (Gene ID: 78971; GenBank: U6-shRNA[rBirc3]3), together with a scrambled control sequence, TTCTCCGAACGTGTCACGT, were synthesized and cloned into the GV859 (U6-MCS-SV40 polyA) and KV815 (MCS-SV40 polyA) adeno-associated virus (AAV) vectors by Shanghai GeneChem Co., Ltd. The cloning sites were AgeI and EcoRI. The inserted fragment was verified by PCR and Sanger sequencing using the following primers: forward primer K25F1953-P1, 5′-ATCAGCGAGCTCTAGTTAATTAATCGAGCGGC-3′; and reverse primer K25F1953-P2, 5′-GTGCATACCTGCGGACCGGTAAAAAGCCAAGT-3′. The amplified fragment size was 1076 bp, and positive clones were identified based on a 1217 bp PCR product. The recombinant sequence was further confirmed using BGH-R, 5′-GACACCTACTCAGACAATGCG-3′, and SV40-Pater, 5′-TTGCAGCTTATAATGGTTACAAAT-3′, primers.

Recombinant AAV9 particles were generated by co-transfecting AAV-Birc3-shRNA or AAV-control plasmids with pHelper and pRepCap plasmids into HEK293T cells using Lipofectamine™ 2000 reagent (Invitrogen, USA). At 72 h after transfection, AAV particles were harvested and purified by iodixanol gradient ultracentrifugation, followed by concentration using Amicon Ultra-15 centrifugal filters. Viral titers were determined by qPCR-based quantification and expressed as genome copies per milliliter (GC/mL). All viral preparations were diluted in 0.001% Pluronic F-68 solution, also known as Poloxamer 188 (Caisson Laboratories, USA), before use.

Immediately after reperfusion, 30 μL of AAV9-Birc3-shRNA or control AAV9 at a titer of 1 × 10¹² GC/mL was injected into three sites in the left ventricular anterior wall surrounding the infarct region using a 33-gauge Hamilton microsyringe. After injection, the chest was closed, and the animals were allowed to recover under standard conditions.

To further determine whether apoptosis inhibition based on AAV-shBirc3 intervention could protect failing rat hearts after ischemia/reperfusion injury, an additional group of rats received Z-VAD-FMK treatment at 1 mg/kg (HY-16658B, MCE, USA) for 4 weeks. Z-VAD-FMK was dissolved in 10% DMSO, diluted with sterile saline, and administered by tail vein injection.

### 2.9 Western blotting

Total proteins were extracted from rat myocardial tissues or H9c2 cells using RIPA lysis buffer (G2002, Servicebio, Wuhan, China) containing PMSF (ST506-2, Beyotime, Shanghai, China). Protein concentrations were determined using a BCA Protein Assay Kit (CW0014, Kangwei, Jiangsu, China). Equal amounts of protein (30-50 μg) were separated by SDS-PAGE (G2037, Servicebio, Wuhan, China) and transferred onto 0.2 μm PVDF membranes (1620177, Bio-Rad, USA). The membranes were blocked with 5% non-fat milk (232100, BD, USA) for 1 h at room temperature, and then incubated overnight at 4 °C with the following primary antibodies: GSDMD (full-length and N-terminal GSDMD) (A24476, ABclonal, Wuhan, China), pMLKL (AP1173, ABclonal, Wuhan, China), Cleaved CASP3 (#9664, Cell Signaling Technology, USA), BIRC3 (A0833, ABclonal, Wuhan, China), ZBP1 (13285-1-AP, Proteintech, Wuhan, China), β-Actin (20536-1-AP, Proteintech, Wuhan, China).

After washing with TBST, the membranes were incubated with HRP-conjugated anti-rabbit IgG secondary antibody (#7074, Cell Signaling Technology, USA) for 1 h at room temperature. Protein bands were visualized using an ECL substrate kit (E412-02, Vazyme, Nanjing, China) with a ChemiDoc XRS+ imaging system (Bio-Rad, USA). Band intensities were quantified using ImageJ software (National Institutes of Health, USA), and normalized to β-actin.

### 2.10 Bioinformatics analysis

#### 2.10.1 Establishment of the background PPI network for PANoptosis

Relevant genes associated with PANoptosis were identified by interrogating the GeneCards database (https://www.genecards.org/) using the keyword “PANoptosis” (15). Subsequently, the STRING database (https://string-db.org/) was employed to retrieve proteins exhibiting high-confidence interactions (confidence score threshold set at 0.900) with these initial candidates (16). To determine the programmed cell death (PCD) pathways in which these proteins are implicated, additional searches were conducted in the GeneCards database using the keywords “Apoptosis,” “Pyroptosis,” and “Necroptosis.” Finally, the background protein-protein interaction (PPI) network for PANoptosis was visualized using Cytoscape software, enabling the identification of genes concurrently involved in all three aforementioned PCD pathways.

#### 2.10.2 GEO dataset processing

All publicly available datasets for ischemic heart failure with sample sizes exceeding 15 were compiled from the GEO database (https://www.ncbi.nlm.nih.gov/geo/), including GSE57338, GSE5406, GSE16499, GSE21610, GSE116250, and GSE161472. GSE57338 was designated as the primary analysis cohort and randomly partitioned into a 70% training set and a 30% internal validation set (17–22). The remaining datasets served as external validation cohorts. Normalization of all datasets was performed utilizing the sva package in R software version 4.4.2 (all bioinformatics analyses were conducted in R software 4.4.2, as subsequently indicated).

#### 2.10.3 Functional enrichment analysis and PANoptosis Bayesian regulatory network

Biological functions associated with PANoptosis-related genes were elucidated through Reactome pathway enrichment analysis. Subsequently, the Bayesian regulatory network for the top 10 enriched Reactome pathways was constructed using the CBNplot package and integrated via Cytoscape software (23).

#### 2.10.4 Machine learning approaches for identifying key PANoptosis genes

This study comprehensively employed twelve distinct machine learning methodologies to delineate pivotal PANoptosis-associated genes in the context of heart failure. The algorithms utilized encompassed Elastic Net, Lasso Regression, Ridge Regression, Stepwise Logistic Regression, Support Vector Machine, Linear Discriminant Analysis, Generalized Linear Model Boosting, Partial Least Squares Regression integrated with Generalized Linear Model, Random Forest, Gradient Boosting Machine, Extreme Gradient Boosting, and Naive Bayes.

#### 2.10.5 Immune infiltration analysis and cell death scoring

Given that PANoptosis represents an inflammatory programmed cell death process, this study further employed both the CIBERSORT and ssGSEA algorithms to perform immune infiltration analysis and cell death scoring within the training set. A correlation heatmap was subsequently constructed to visualize the relationships between key genes and the resulting scoring metrics.

#### 2.10.6 Single-cell perspective on cell death scoring, pseudotime analysis, and virtual gene knockout

The dataset GSE247468 was selected for single-cell analysis in this study (24). Following dimensionality reduction and clustering, cells were annotated into distinct types based on prior biological knowledge. Cell death scoring was subsequently performed using the singscore algorithm. Pseudotime trajectory analysis was then conducted with Monocle3 to identify PANoptosis-related genes demonstrating significant dynamic expression changes and to elucidate key regulatory factors throughout the process. Finally, virtual gene knockout analysis was carried out using the scTenifoldKnk package to investigate alterations in gene expression patterns following the knockdown of pivotal genes (25).

### 2.11 Statistical analysis

Data are presented as mean ± standard deviation (SD). Unless otherwise indicated, default parameters were employed for all bioinformatics analyses. Inter-group differences were assessed using one-way ANOVA in GraphPad Prism 9.0, P values less than 0.05 were considered statistically significant.

## 3 Results

### 3.1 Immunofluorescence staining and transmission electron microscopy analysis

**Figure 2:**
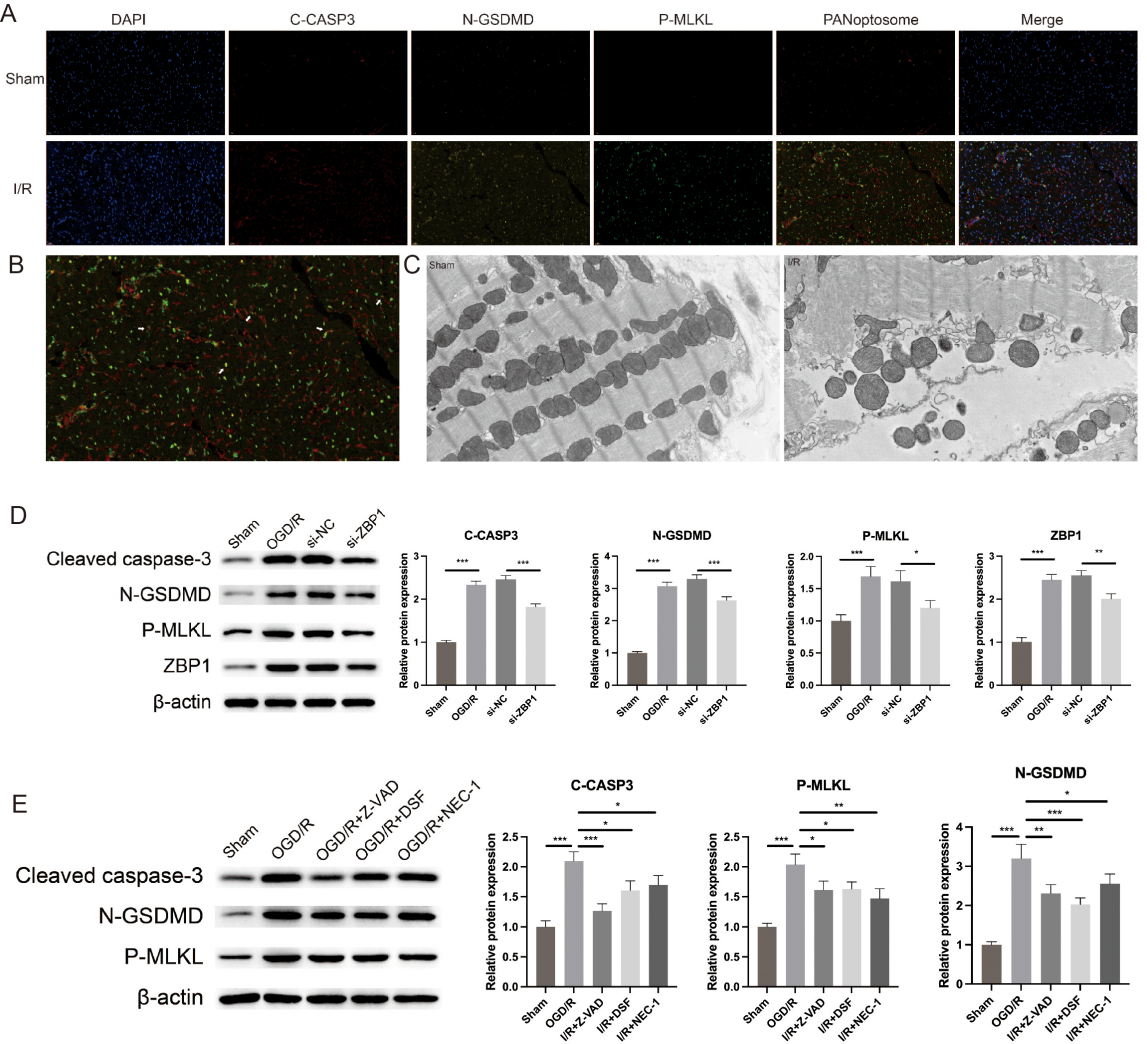
PANoptosis activation in myocardial ischemia-reperfusion injury. **(A)** Immunofluorescence images of C-CASP3, N-GSDMD, and P-MLKL. **(B)** Overlap in the executive proteins of the three PCD types, indicated by white arrows. **(C)** Transmission electron microscopy images. **(D)** Western blot images and quantification of programmed cell death–related biomarkers after silencing PANoptosis-related ZBP1 (n=3). **(E)** Western blot images and quantification of PCD-related biomarkers after treatment with apoptosis, pyroptosis, and necroptosis inhibitors (n=3). *P<0.05, **P<0.01, ***P<0.001.

To investigate the specific modes of cell death in ischemia/reperfusion (I/R)-induced heart failure, we first performed immunofluorescence staining on myocardial tissue sections from rat models, labeling key executioner proteins: C-CASP3 for apoptosis, N-GSDMD for pyroptosis, and p-MLKL for necroptosis. The results showed that I/R injury significantly upregulated the expression of all three PCD executioners (**Figure 2A**). Furthermore, spatial colocalization analysis revealed pronounced overlap in the subcellular distribution of these proteins, suggesting potential physical or functional interactions among them. These findings are consistent with coordinated activation and partial colocalization of apoptotic, pyroptotic, and necroptotic executioner proteins, supporting the presence of PANoptosis-like cell death in cardiomyocytes. (**Figure 2B**).

To further investigate the forms of cell death, TEM was conducted on myocardial tissues. In the Sham group, cardiomyocytes exhibited typical ultrastructural integrity: mitochondria were localized at both longitudinal ends of the sarcomeres, aligned parallel to and uniformly distributed among the myofibrils. Mitochondria were abundant with intact outer and inner membranes, electron-dense matrices, and well-organized cristae. Myofibrils were neatly arranged with clearly visible Z-lines and alternating light and dark bands. In contrast, the I/R group exhibited significantly swollen mitochondria with irregular or ruptured outer membranes, disintegrated or blurred cristae, and vacuolated matrices, indicative of mitochondrial autophagy. Additionally, myofibrillar disarray, Z-line obscuration or loss, and loss of sarcomeric definition were observed. The cell membrane showed blebbing and rupture. Collectively, these findings indicated that I/R induced mixed morphological features of apoptosis, pyroptosis, and necroptosis, suggesting the occurrence of PANoptosis (**Figure 2C**).

### 3.2 Functional validation of PANoptosis using siRNA and inhibitors

To investigate whether myocardial injury induced by I/R is specifically mediated by PANoptosis rather than by individual PCD pathways acting independently, this study utilized siRNA to inhibit the expression of ZBP1—a key sensor of PANoptosis—in an OGD/R-induced H9C2 cell injury model. Western blot analysis revealed that the OGD/R model group exhibited a significant upregulation of ZBP1, accompanied by marked increases in the levels of key PCD marker proteins: C-CASP3 for apoptosis, N-GSDMD for pyroptosis, and P-MLKL for necroptosis. Following ZBP1 knockdown, the expression of all three PCD markers decreased simultaneously, indicating that OGD/R activates PANoptosis, which integrates features of multiple PCD modalities, rather than activating a single form of programmed cell death (**Figure 2D**).

However, it should be noted that ZBP1 is only one of several sensors involved in PANoptosis; other molecules such as RIPK1 and NLRP12 have also been identified as regulators capable of mediating PCD in this context (26, 27). Therefore, silencing ZBP1 alone is insufficient to completely suppress PANoptosis activation, underscoring the need for further investigation into the regulatory mechanisms of PANoptosis.

Subsequently, the OGD/R-induced injury model was treated with the apoptosis inhibitor Z-VAD-FMK, the pyroptosis inhibitor disulfiram, and the necroptosis inhibitor necrostatin-1. Western blot analysis demonstrated that Z-VAD-FMK not only alleviated apoptosis but also partially attenuated pyroptosis and necroptosis. Similar cross-inhibitory effects were observed following treatment with disulfiram and necrostatin-1 (**Figure 2E**). These findings suggest the presence of mutual regulation among different PCD pathways, likely mediated through the overarching mechanism of PANoptosis.

Collectively, these results confirm that myocardial injury induced by I/R or OGD/R involves PANoptosis, a process supported by cross-talk among apoptosis, pyroptosis, and necroptosis.

### 3.3 Exploring effective strategies for PANoptosis inhibition

To investigate potential strategies for inhibiting PANoptosis, we evaluated various interventional approaches in an OGD/R-induced H9C2 cell injury model, including the application of individual inhibitors, dual combinations, and a triple combination of inhibitors targeting distinct PCD pathways.

**Figure 3:**
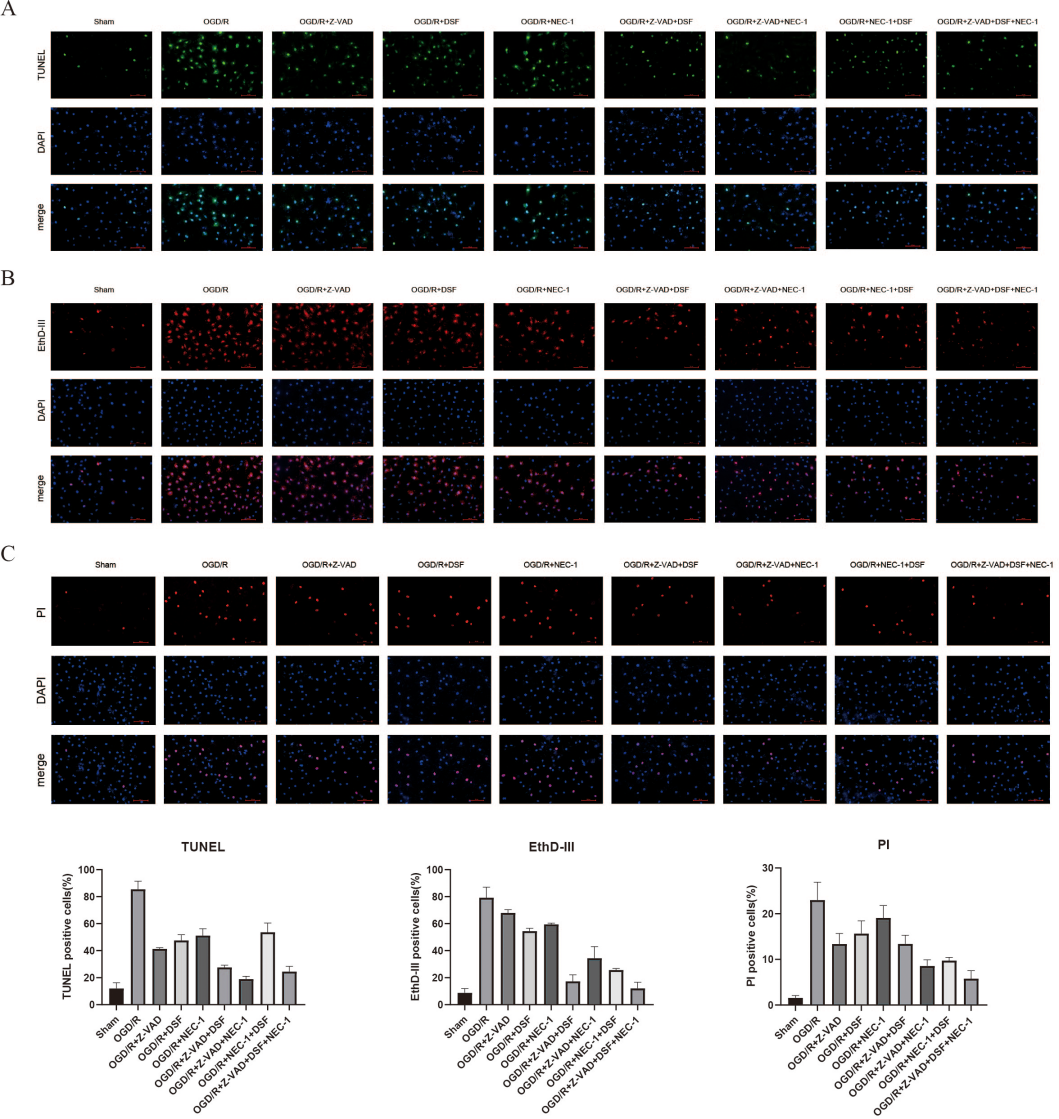
Mortality rates of different PCD types. **(A–C)**: Representative images and quantitative results of TUNEL staining, EthD-III staining, and PI staining, respectively, following treatment with apoptosis, pyroptosis, and necroptosis inhibitors (n=5).

Cell death was assessed using TUNEL staining to evaluate apoptosis (**Figure 3A**), EthD-III staining for pyroptosis (**Figure 3B**), and propidium iodide (PI) staining to monitor necroptosis (**Figure 3C**). The results revealed that inhibition of one specific PCD pathway led to a concomitant reduction in other forms of PCD. Furthermore, combined inhibitor treatments demonstrated superior efficacy compared to individual inhibitors. Notably, however, the triple inhibitor combination did not yield additional benefit compared to dual inhibitor strategies.

These findings demonstrate that within the OGD/R-induced H9C2 cell injury model, there exists close cross-talk among the three programmed cell death pathways: apoptosis, pyroptosis, and necroptosis. Inhibition of any single pathway resulted in the simultaneous attenuation of the other two forms of cell death, confirming that these pathways form an interconnected regulatory network under the PANoptosis framework. Although dual-pathway inhibition exhibited superior synergistic protective effects compared to single inhibitors, the triple-pathway combination did not yield additional benefits, suggesting the potential existence of a saturation effect or core pathway bottleneck in PANoptosis regulation. This insight provides an important basis for optimizing intervention strategies targeting PANoptosis in myocardial ischemia-reperfusion injury.

### 3.4 Construction of the PANoptosis regulatory network

To elucidate the complex regulatory mechanisms underlying PANoptosis, we initiated our investigation by retrieving PANoptosis-associated genes from the GeneCards and STRING databases and constructing a protein-protein interaction (PPI) network. Using the keyword “PANoptosis” in GeneCards, we identified 23 related proteins. Subsequently, the STRING database was employed to expand this set by including proteins with high-confidence interactions (confidence threshold set at 0.900) with the initial candidates. Integrating results from both databases, we identified 137 proteins potentially associated with PANoptosis.

**Figure 4:**
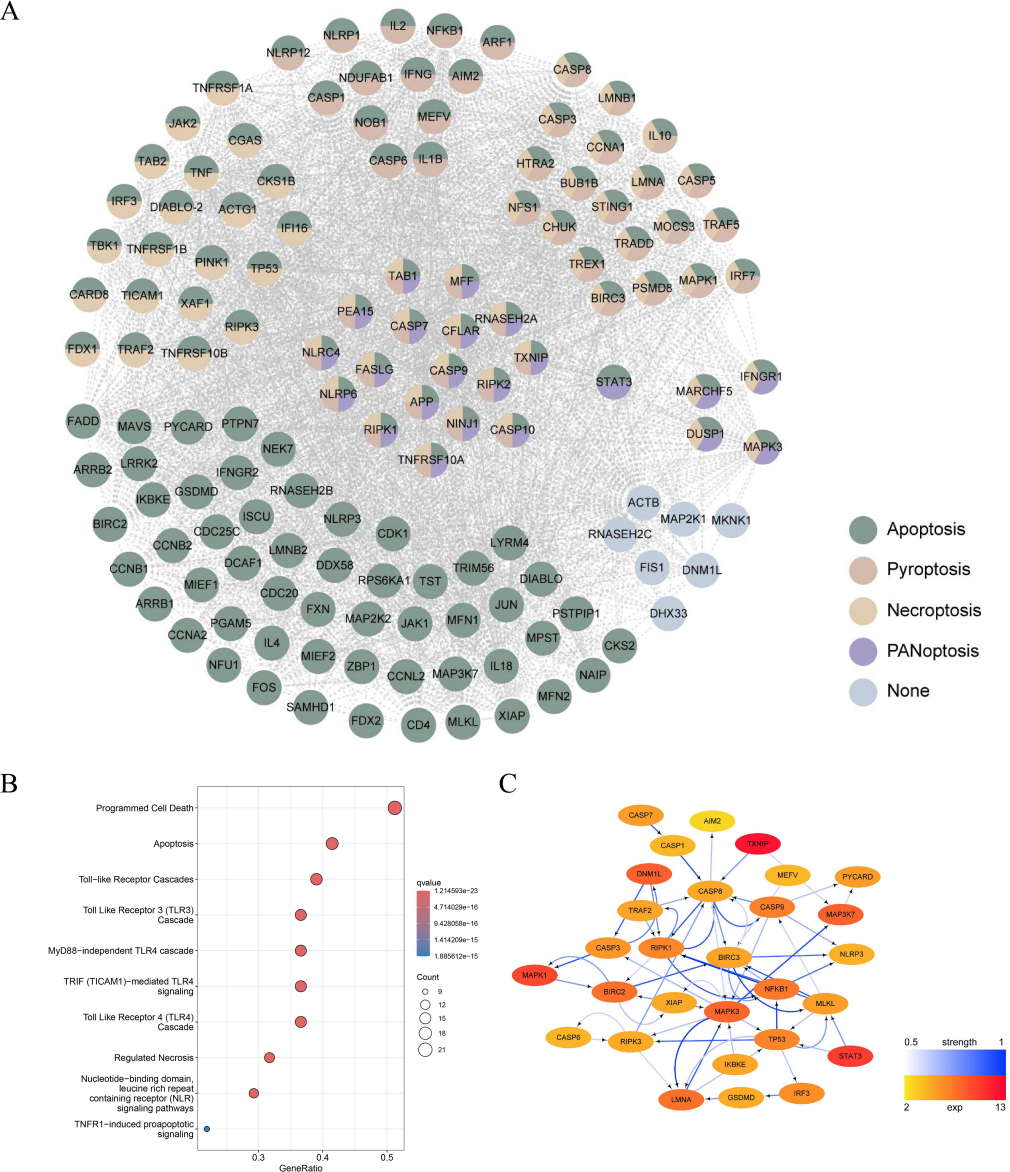
Protein–protein interaction and pathway analysis of PANoptosis-related proteins. **(A)** PPI network of PANoptosis-related proteins within the corresponding programmed cell death pathways. **(B)** Reactome pathway enrichment analysis. **(C)** Bayesian regulatory network of PANoptosis-related genes.

To determine the involvement of these proteins in specific PCD pathways, we further queried GeneCards using the keywords “apoptosis,” “pyroptosis,” and “necroptosis.” The comprehensive PPI network for PANoptosis was visualized using Cytoscape (**Figure 4A**).

Analysis of the PPI network revealed 42 proteins that have been experimentally validated to be associated with PANoptosis or implicated in all three PCD pathways. To investigate their biological functions, we performed Reactome pathway enrichment analysis. The results indicated significant enrichment in pathways related to programmed cell death, apoptosis, Toll-like receptor (TLR) cascades, TLR3 cascades, and MyD88-independent TLR4 cascades (**Figure 4B**).

To further decipher the regulatory relationships among these 42 proteins in the context of heart failure, we constructed a Bayesian regulatory network using the CBNplot tool. The largest available heart failure dataset, GSE57338, was retrieved from the GEO database and randomly divided into training and validation sets at a 7:3 ratio. Using expression profiles from heart failure samples in the training set, we generated a Bayesian regulation network encompassing the top 10 enriched Reactome pathways and integrated it via Cytoscape (**Figure 4C**).

### 3.5 Bioinformatic analysis of heart failure transcriptomes

In this study, we comprehensively employed twelve distinct machine learning algorithms—Elastic Net, Lasso Regression, Ridge Regression, Stepwise Logistic Regression, Support Vector Machine, Linear Discriminant Analysis, Generalized Linear Model Boosting, Partial Least Squares Regression with Generalized Linear Model, Random Forest, Gradient Boosting Machine, Extreme Gradient Boosting, and Naive Bayes—to delineate pivotal PANoptosis-associated genes within the context of heart failure. The GSE57338 dataset was utilized, with 70% of its samples allocated to the training set and the remaining 30% designated as the validation set. Furthermore, we aggregated all publicly accessible ischemic heart failure datasets with sample sizes exceeding 15, including GSE5406, GSE16499, GSE21610, GSE116250, and GSE161472, which served as external validation cohorts.

**Figure 5:**
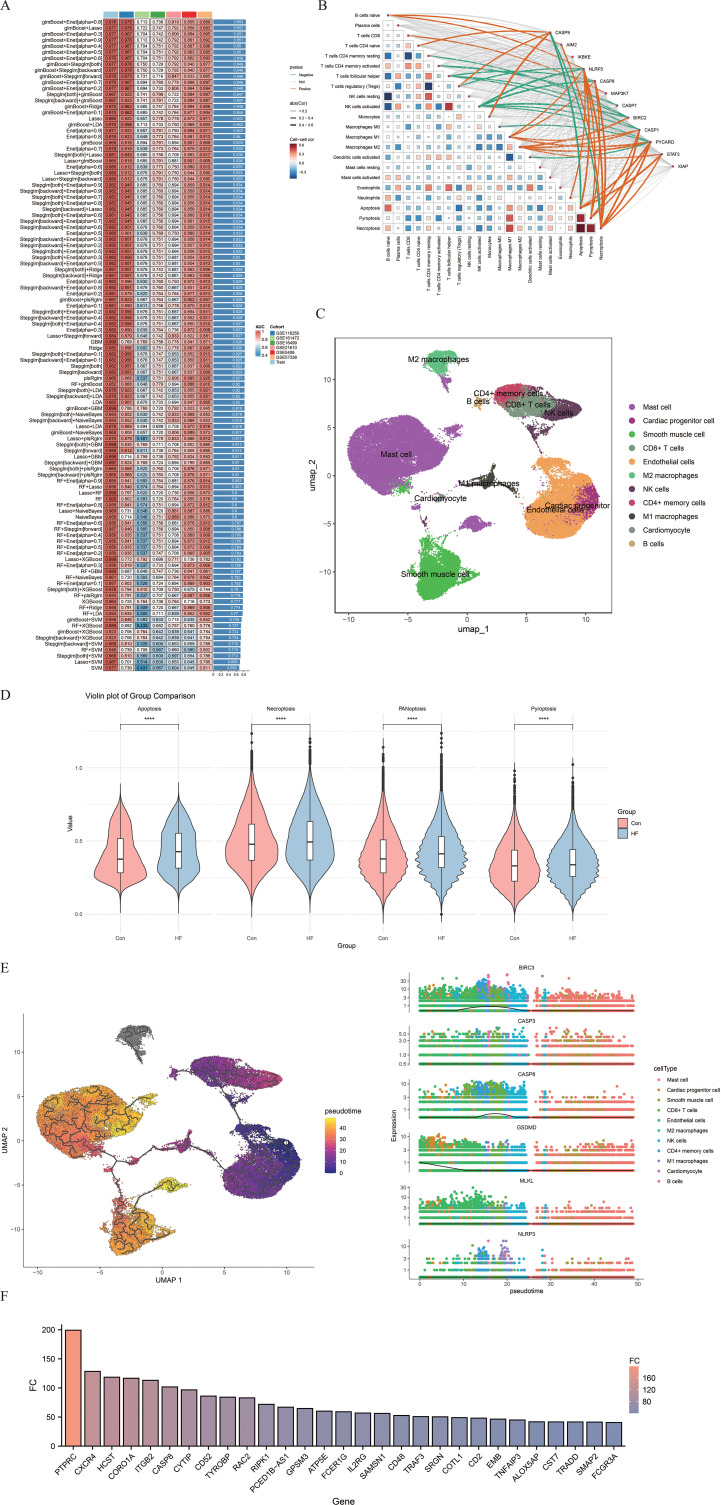
Transcriptomic and single-cell sequencing analyses of PANoptosis in heart failure. **(A)** Key PANoptosis-related genes identified using combined machine learning algorithms. **(B)** Immune infiltration analysis, cell death scoring, and correlation heatmap of key genes and scoring metrics in the training set using CIBERSORT and ssGSEA algorithms. **(C)** Cell type identification in the GSE247468 dataset. **(D)** Cell death scores of cardiomyocytes. **(E)** Pseudotime expression analysis. **(F)** Gene expression changes after virtual knockout of BIRC3. ****P<0.0001.

Analytical results demonstrated that the integrative model combining glmBoost and Enet (alpha = 0.8) achieved the highest mean accuracy (AUC = 0.853) across all datasets, with each individual dataset attaining an accuracy above 0.7 (**Figure 5A**). Utilizing this model, we identified 12 genes critically implicated in PANoptosis, namely CASP9, AIM2, IKBKE, NLRP3, CASP8, MAP3K7, CASP7, BIRC2, CASP1, PYCARD, STAT3, and XIAP.

Given that PANoptosis constitutes an inflammatory programmed cell death process, we further conducted immune infiltration analysis and cell death scoring on the training set using the CIBERSORT and ssGSEA algorithms, and generated a correlation heatmap illustrating associations between key genes and scoring metrics (**Figure 5B**). The heatmap revealed pronounced positive correlations among apoptosis, pyroptosis, and necroptosis, implying shared molecular mechanisms and intricate regulatory networks, i.e., PANoptosis. Among immune cell subtypes, M1 macrophages were positively associated with PANoptosis-related scores, whereas most other immune cell types showed inverse correlations. Among the key genes, CASP8, NLRP3, CASP1, and PYCARD were positively associated with PANoptosis-related metrics, suggesting their potential involvement in cellular responses to death signals.

### 3.6 Elucidation of PANoptosis regulatory mechanisms through single-cell analysis

The dataset GSE247468, comprising two normal samples and four heart failure samples, was selected for single-cell analysis. We identified 11 distinct cell types, including mast cells, cardiac progenitor cells, smooth muscle cells, CD8+ T cells, endothelial cells, M2 macrophages, NK cells, CD4+ memory cells, M1 macrophages, cardiomyocytes, and B cells (**Figure 5C**).

Subsequently, cell death scoring was performed using the singscore algorithm. The results demonstrated significantly elevated scores for apoptosis, pyroptosis, necroptosis, and PANoptosis in heart failure samples compared to normal controls, indicating heightened activity of programmed cell death pathways in heart failure (**Figure 5D**).

Pseudotime trajectory analysis was conducted using Monocle3 to identify dynamically expressed PANoptosis-related genes and explore key regulatory factors. The analysis revealed that BIRC3 and CASP8 exhibited coordinated expression dynamics throughout pseudotime. Given their previously identified mutual regulatory relationships in the PANoptosis Bayesian network, these findings suggest their potential role as core regulators of PANoptosis (**Figure 5E**).

Finally, virtual knockout of BIRC3 was simulated using scTenifoldKnk, which revealed profound alterations in CASP8 expression levels (**Figure 5F**). This further supports the functional interplay between BIRC3 and CASP8 within the PANoptosis regulatory framework.

### 3.7 Directional regulation of PANoptosis by BIRC3 via modulation of CASP8

Through Bayesian network and pseudotime trajectory analyses, BIRC3 and CASP8 were identified as potential key regulators of PANoptosis. To further elucidate their mechanistic roles, experimental validation was conducted in an OGD/R-induced H9C2 cell model.

**Figure 6:**
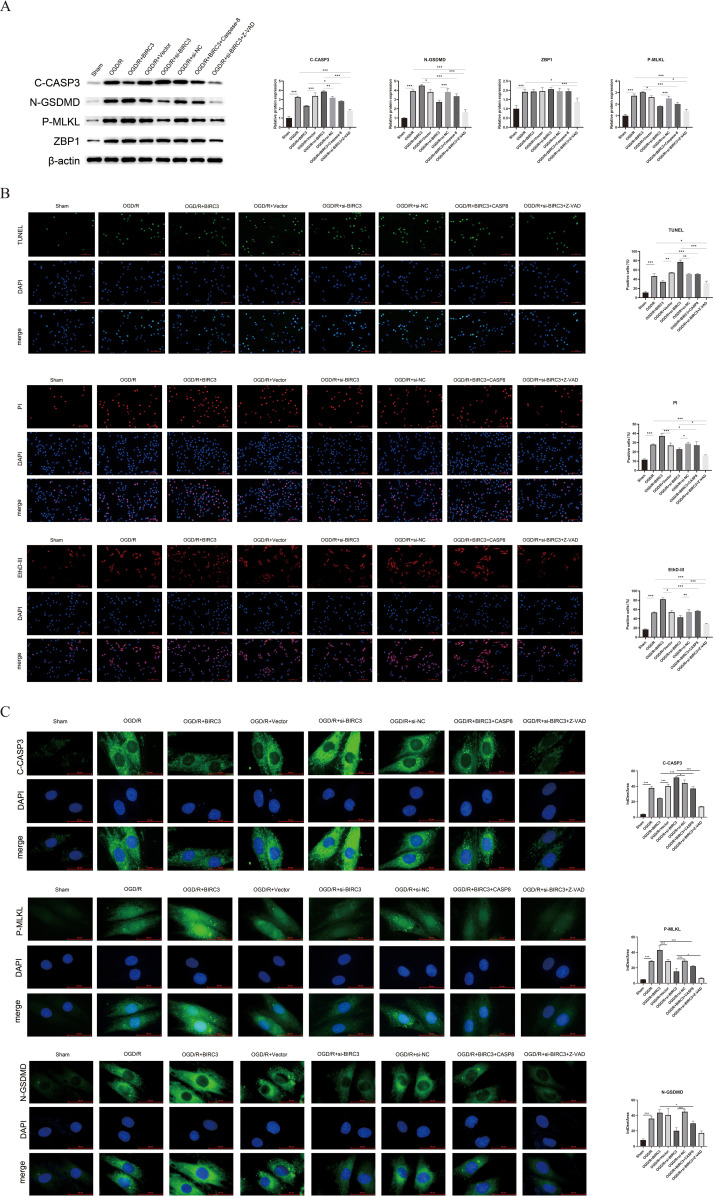
Experimental validation of BIRC3 and CASP8 in PANoptosis regulation. **(A)** Western blot analysis of the effects of BIRC3 modulation on cell death patterns in OGD/R-treated H9C2 cells (n=3). **(B)** Staining analysis of changes in apoptosis, pyroptosis, and necroptosis after BIRC3 up- or downregulation (n=5). **(C)** Immunofluorescence analysis of the effects of BIRC3-CASP8 on the expression of PCD executive proteins (n=5). *P<0.05, **P<0.01, ***P<0.001.

Western blotting (**Figure 6A**), staining (**Figure 6B**), and immunofluorescence (**Figure 6C**) analyses consistently demonstrated that upregulation of BIRC3 shifted the cell death pattern from PANoptosis toward reduced apoptosis, with increased proportions of pyroptosis and necroptosis. Conversely, BIRC3 knockdown enhanced apoptosis while diminishing pyroptosis and necroptosis, underscoring the pivotal role of BIRC3 in modulating PANoptosis directionality.

Bayesian network analysis suggested potential regulatory interplay between BIRC3 and CASP8. Co-upregulation experiments revealed that CASP8 overexpression counteracted the anti-apoptotic effects induced by BIRC3 upregulation, indicating that BIRC3 may exert its regulatory influence on PANoptosis by targeting CASP8. Furthermore, the combination of BIRC3 silencing and apoptosis inhibitor treatment markedly suppressed PANoptosis and overall cell death.

**Figure 7:**
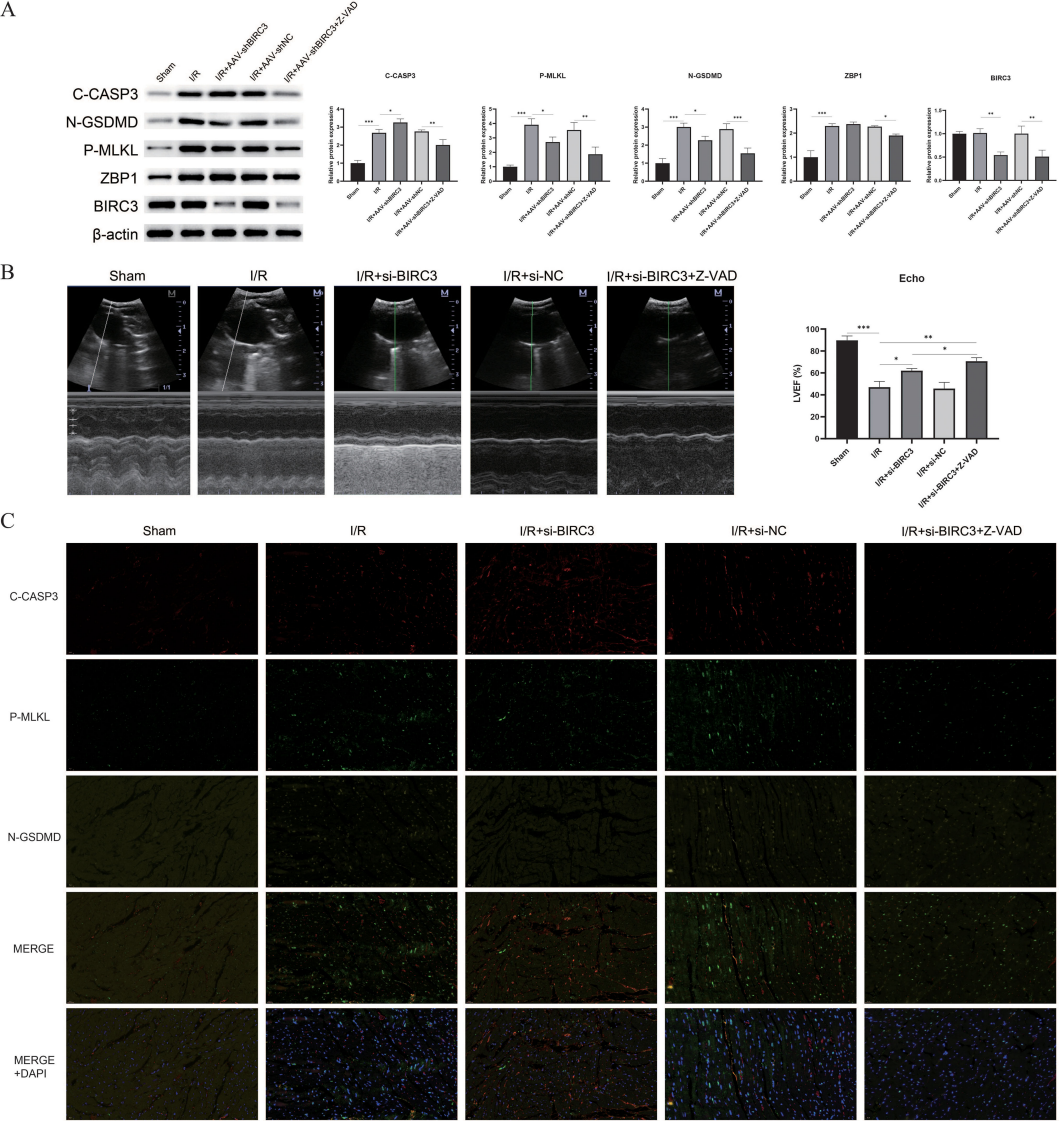
In vivo validation of BIRC3 in PANoptosis regulation. **(A)** Western blot analysis of the effects of BIRC3 knockdown and apoptosis inhibition on PANoptosis in heart tissue (n=3). **(B)** Echocardiographic assessment of LVEF improvement following combined BIRC3 silencing and Z-VAD-FMK treatment (n=5). **(C)** Immunofluorescence analysis of reduced PANoptosis occurrence after the combined intervention. *P<0.05, **P<0.01, ***P<0.001.

In animal models, Western blot results corroborated the cellular findings (**Figure 7A**). BIRC3 knockdown promoted a shift toward apoptosis while reducing pyroptosis and necroptosis. The addition of an apoptosis inhibitor following BIRC3 silencing significantly attenuated PANoptosis incidence, aligning with cellular observations and reinforcing BIRC3’s central role in PANoptosis regulation.

Echocardiographic assessments demonstrated that the combined intervention of BIRC3 silencing and the apoptosis inhibitor Z-VAD-FMK effectively restored left ventricular ejection fraction (LVEF) in heart failure rats (**Figure 7B**).

Immunofluorescence analysis further confirmed that this combined treatment robustly reduced PANoptosis occurrence (**Figure 7C**). These findings not only emphasize the significance of BIRC3 in PANoptosis regulation but also provide experimental support for developing therapeutic strategies targeting PANoptosis. Collectively, these results suggest that modulating BIRC3 expression in conjunction with apoptosis inhibition may represent a promising intervention for controlling PANoptosis.

## 4 Discussion

Heart failure (HF) remains a predominant contributor to global morbidity and mortality, driven by complex pathological alterations including myocardial ischemia/reperfusion (I/R) injury (28). While timely reperfusion is critical for salvage, it paradoxically exacerbates oxidative stress and inflammation, triggering massive cardiomyocyte loss (29). This cell death is not merely an endpoint but a driver of clinical outcomes (30). However, the concurrent activation of multiple programmed cell death (PCD) pathways complicates traditional therapeutic approaches (5). When cardiomyocytes damaged by severe ischemia are destined to die, a pragmatic therapeutic strategy may not be to suppress death at all costs, but to minimize the collateral damage it causes. Therefore, elucidating the mechanisms governing the “mode” of cell death offers novel insights for HF treatment.

Since the conceptualization of PANoptosis, establishing a standardized framework—including its occurrence, potential PANoptosome assembly, and key regulatory mechanisms—has been a priority (31). In this study, we provide evidence that myocardial I/R injury is associated with coordinated activation and partial colocalization of apoptotic (C-CASP3), pyroptotic (N-GSDMD), and necroptotic (p-MLKL) executioner proteins. Together with the observed mixed ultrastructural features, these findings support the presence of PANoptosis-like cell death in the myocardium. However, because direct biochemical assays such as co-immunoprecipitation or proximity ligation analysis were not performed, the current data do not definitively demonstrate the assembly of a specific PANoptosome complex. Rather, our findings suggest the potential involvement of PANoptosome-related pathway activation during myocardial I/R injury.

Through bioinformatic profiling, we mapped the molecular landscape of cardiac PANoptosis. The enrichment of 137 PANoptosis-associated genes in PRR signaling and TNFR1 pathways highlights the intrinsic link between cell death and innate immunity. This aligns with the notion that PANoptosis is not merely a demolition process but a potent immunomodulator. PRRs sense DAMPs released from dying cells, creating a feedback loop that amplifies inflammation—a cycle that is particularly destructive in the non-regenerative heart (32–34). Immune infiltration analysis further indicated that M1 macrophages, which drive proinflammatory responses, were positively correlated with PANoptosis (35–37), suggesting that lytic cell death may recruit and activate these immune cells to exacerbate tissue damage.

Mechanistically, our study identifies the BIRC3-CASP8 axis as the pivotal molecular switch of the PANoptotic spectrum. Traditionally, BIRC3 is viewed as a cytoprotective agent that inhibits apoptosis by binding CASP8 (38, 39). However, our findings reveal a paradoxical and maladaptive role for BIRC3 in the context of severe I/R injury. We demonstrate that by strongly inhibiting CASP8, BIRC3 inadvertently blocks the orderly apoptotic pathway, compelling the cell to engage alternative, more destructive effectors—pyroptosis and necroptosis. This “inhibition” results in lytic cell death that triggers severe inflammation. Crucially, we found that silencing BIRC3 releases the brake on CASP8, restoring the default apoptotic program. While the cell still undergoes death, it does so via apoptosis—an immunologically silent process that maintains membrane integrity. This phenotypic shift prevents the release of inflammatory cytokines (e.g., IL-1β) and protects neighboring cardiomyocytes from bystander killing. Thus, our study redefines BIRC3 not just as an apoptosis inhibitor, but as a “phenotypic switch” that dictates the immunogenicity of cell death.

**Figure 8:**
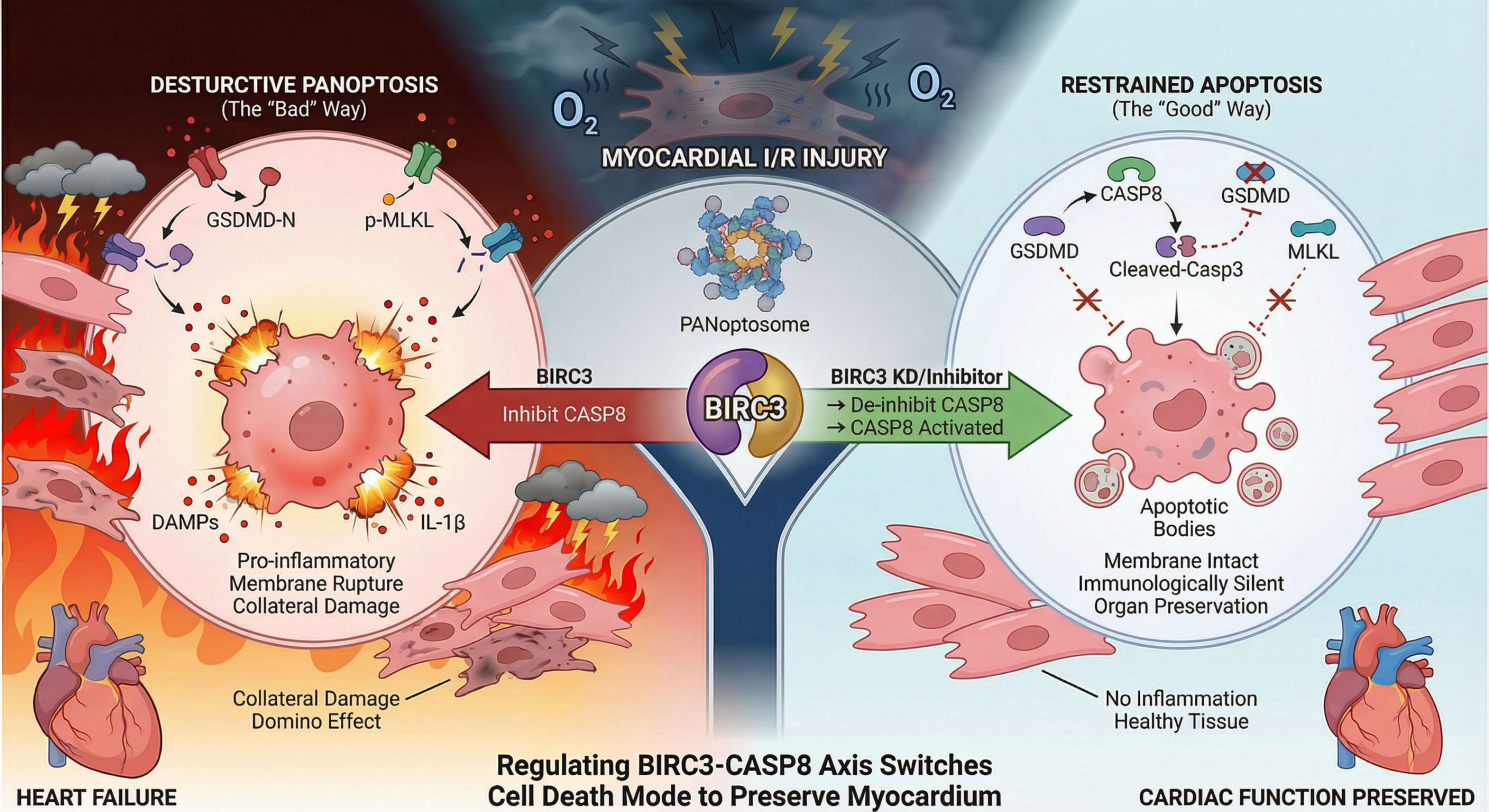
BIRC3-CASP8 axis regulates PANoptosis in myocardial ischemia-reperfusion injury. BIRC3 promotes PANoptosis by inhibiting CASP8 activation, leading to inflammatory cell death and myocardial damage. BIRC3 knockdown or inhibition restores CASP8 activity, shifts cell death toward restrained apoptosis, and preserves cardiac function.

Thus, our findings suggest that BIRC3 may influence not only apoptosis-related signaling but also the inflammatory phenotype of cell death. These observations may have therapeutic implications. Targeting the BIRC3-CASP8 axis may shift cell death toward a less inflammatory apoptotic phenotype and reduce collateral myocardial injury. This approach may help preserve functional myocardial reserve and maintain a more favorable immune microenvironment. The mechanism diagram is shown in **Figure 8**.

This study has several limitations. First, a substantial proportion of the mechanistic experiments were performed in H9c2 cells. Although H9c2 cells are widely used as an *in vitro* model for cardiomyocyte injury, they are cardiomyoblasts and differ from adult cardiomyocytes in several important aspects, including maturation status, metabolic profile, contractile properties, and stress responses. This distinction is particularly relevant when extrapolating the findings to the non-regenerative adult myocardium. In addition, although the mechanisms of PANoptosis were explored using both animal and cellular models, the lack of validation in clinical samples limits the immediate generalizability of the findings, as the heterogeneity of human HF is difficult to fully capture.

Second, the key PANoptosis-related genes in this study were identified using public heart failure datasets. Because acute myocardial ischemia/reperfusion injury and heart failure differ in disease stage and molecular characteristics, this difference may limit the broader applicability of the bioinformatics-derived findings to acute I/R injury. Moreover, the relatively small sample size in the transcriptomic cohort and potential batch effects in public datasets may obscure subtle regulatory networks. In particular, the single-cell analysis was based on a relatively small dataset comprising only two normal samples and four heart failure samples, which may limit the representativeness and generalizability of the single-cell findings. Future studies incorporating primary adult cardiomyocytes, human induced pluripotent stem cell-derived cardiomyocytes, human biopsy samples, larger clinical cohorts, and additional I/R-specific single-cell datasets are warranted to further delineate the clinical applicability of the BIRC3-CASP8 axis.

## 5 Conclusion

In summary, this study suggests that the BIRC3-CASP8 regulatory axis is involved in PANoptosis-related cell death during myocardial ischemia/reperfusion injury. Our findings indicate that PANoptosis may represent a coordinated network of interacting death pathways, in which BIRC3 contributes to the balance between lytic and more restrained cell death phenotypes. These results provide a potential conceptual basis for modulating the mode of cell death to reduce collateral myocardial injury. Future studies are needed to further evaluate the translational potential of BIRC3 modulation and guided cell-death strategies in post-ischemic heart failure.

## Data Availability

The original contributions presented in the study are included in the article/**Supplementary Material**. Further inquiries can be directed to the corresponding authors.
