# Supplementary figures and images for: BIRC3-CASP8 axis orchestrates the PANoptosis spectrum: taming the inflammatory storm to prevent post-ischemic heart failure
Source: Front Immunol. 2026 Jun 29;17:1875226. doi: 10.3389/fimmu.2026.1875226 (PMC13357215; doi:10.3389/fimmu.2026.1875226)

Sham

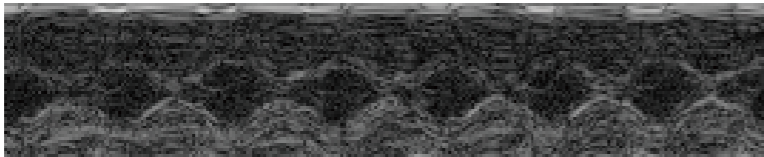

I/R

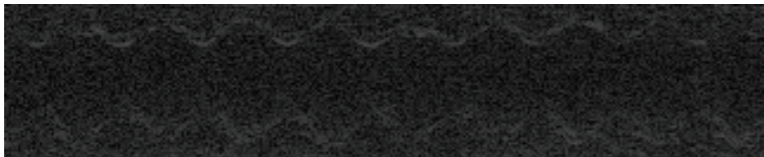

Before LAD ligation

10:

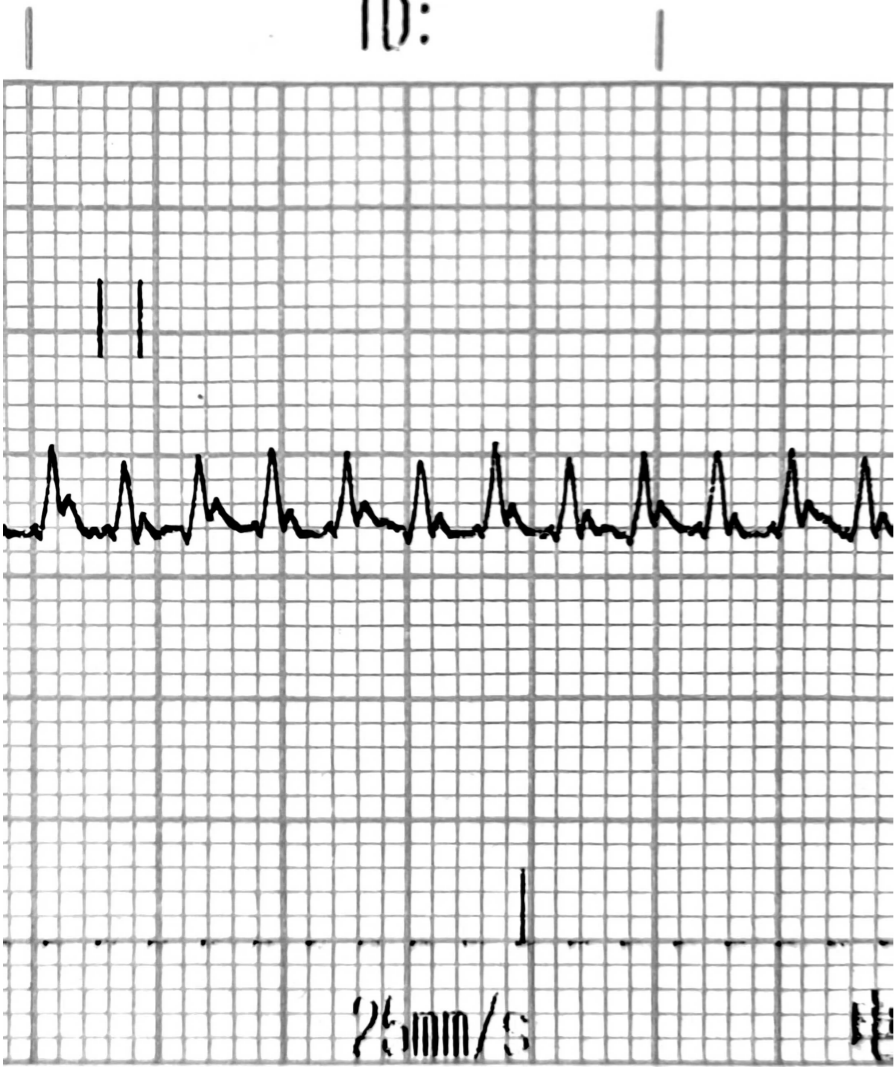

After LAD ligation

10:

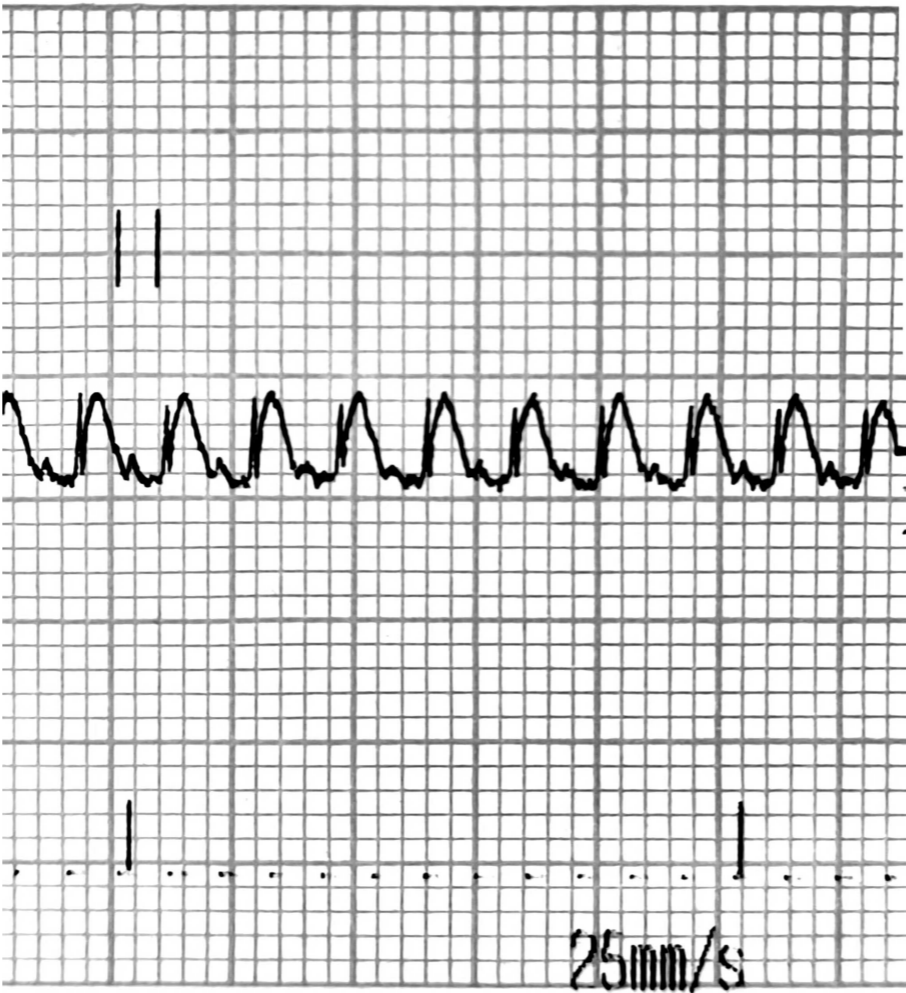

After reperfusion

10:

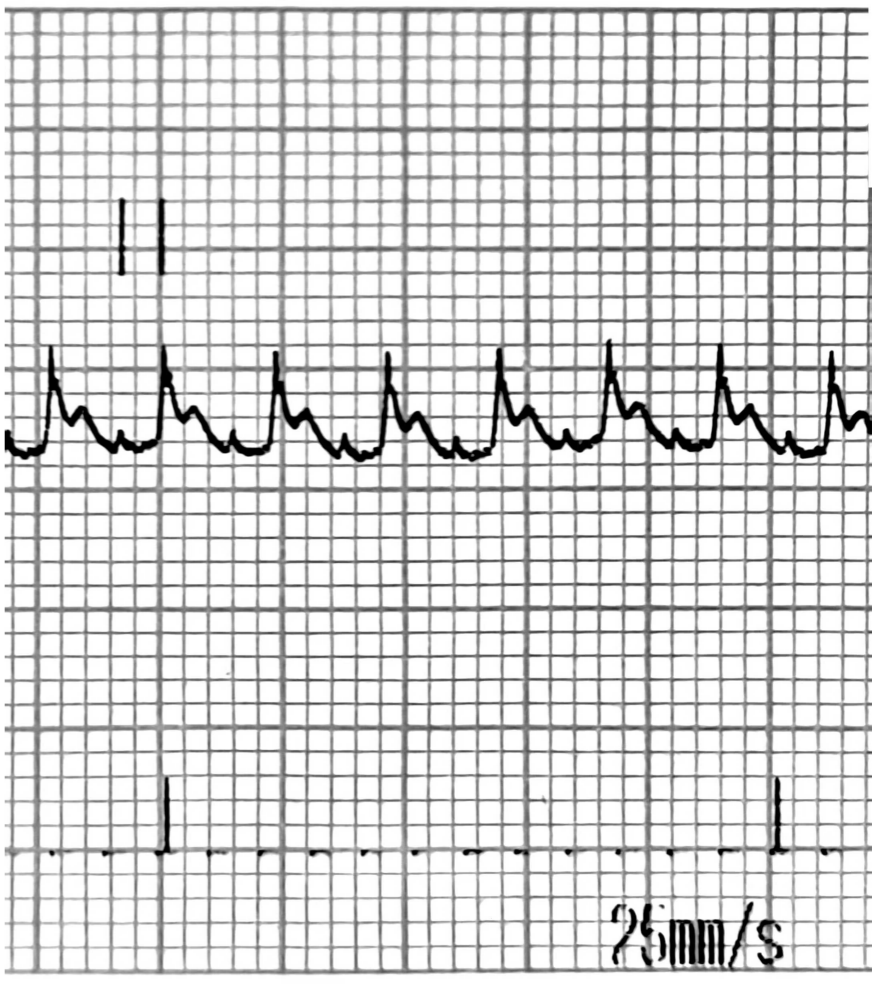

Supplement: Supplementary file 1 [file DataSheet1.pdf]
