# Supplementary material for: BIRC3-CASP8 axis orchestrates the PANoptosis spectrum: taming the inflammatory storm to prevent post-ischemic heart failure
Source: Front Immunol. 2026 Jun 29;17:1875226. doi: 10.3389/fimmu.2026.1875226 (PMC13357215; doi:10.3389/fimmu.2026.1875226)
Supplement: Supplementary file 2 [file DataSheet2.pdf]

Figure2D

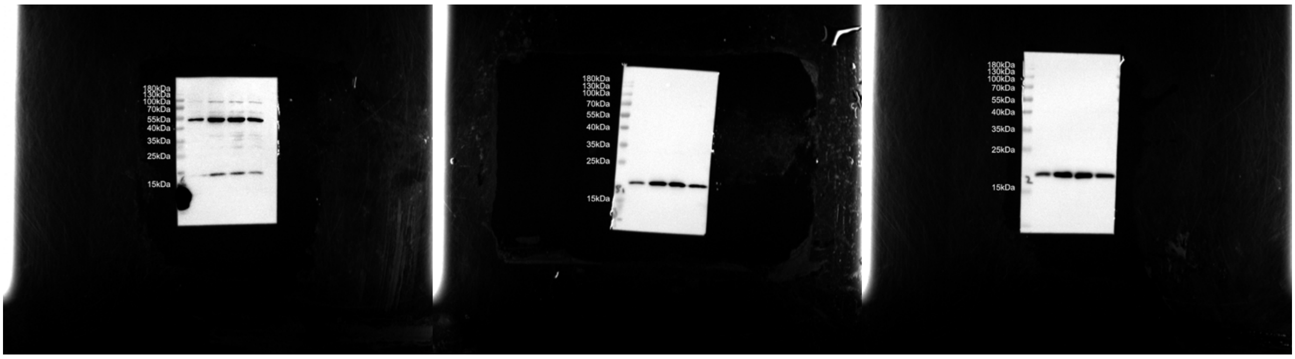

C-CAS3

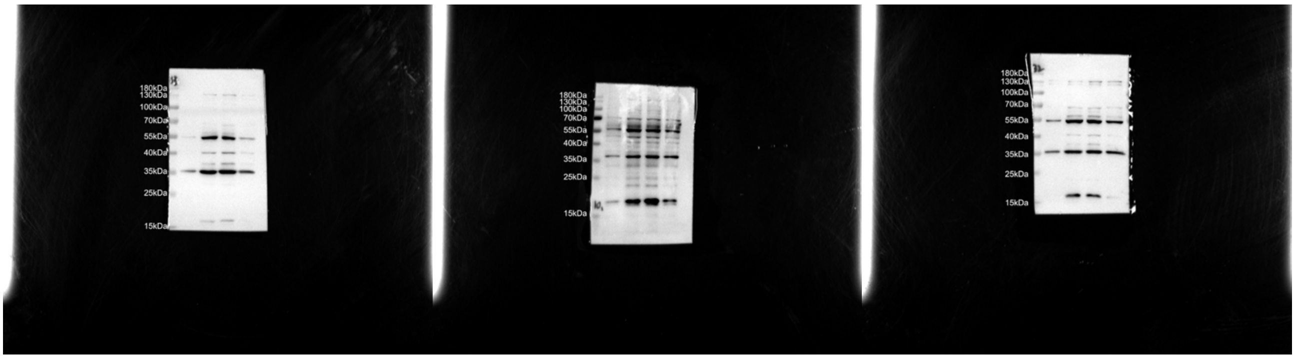

N-GSDMD

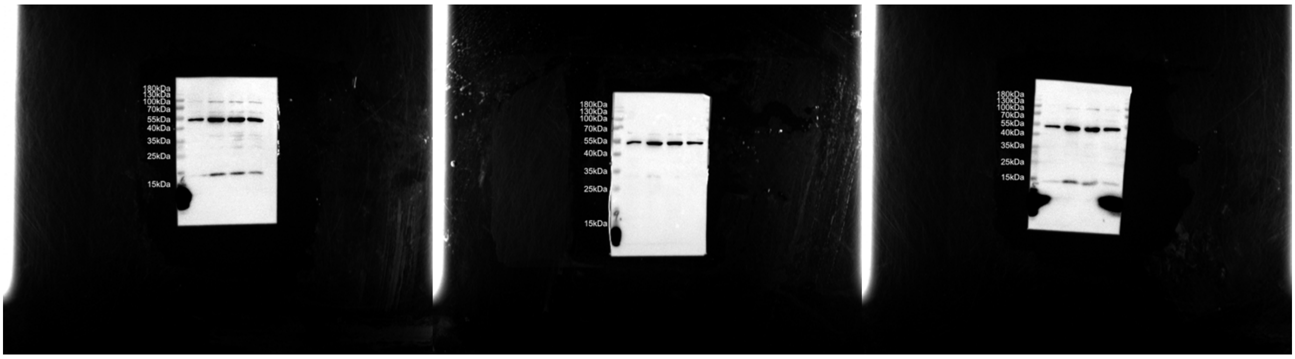

P-MLKL

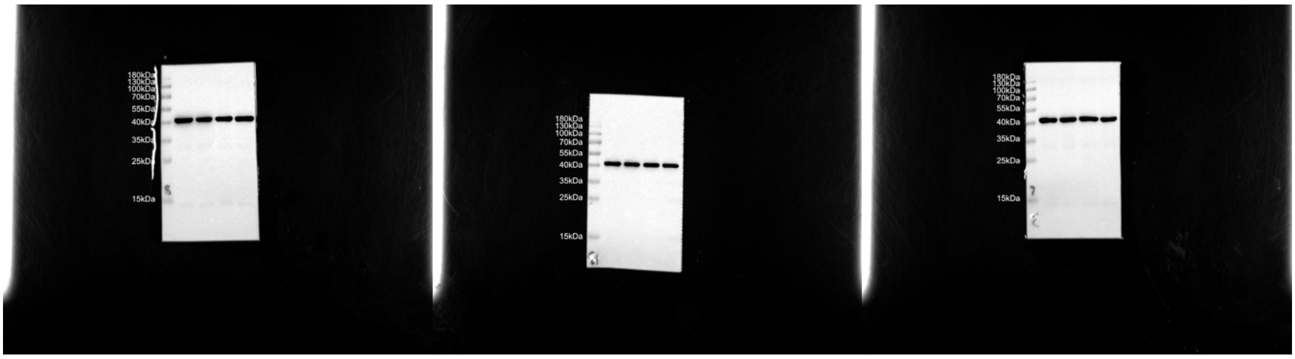

ACTIN

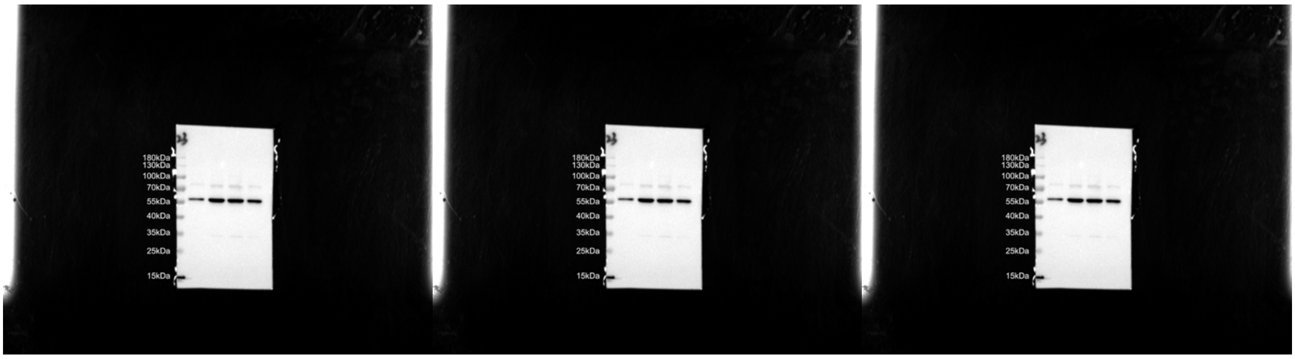

ZBP1

Figure2E

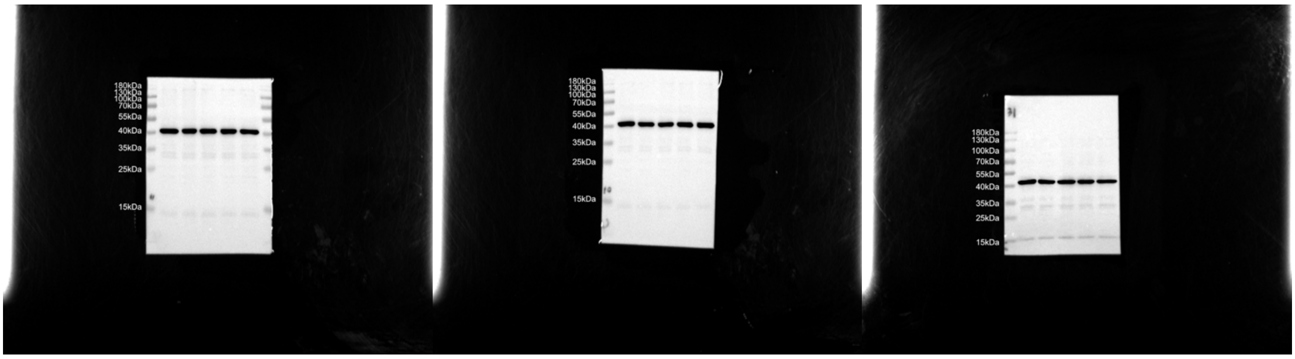

ACTIN

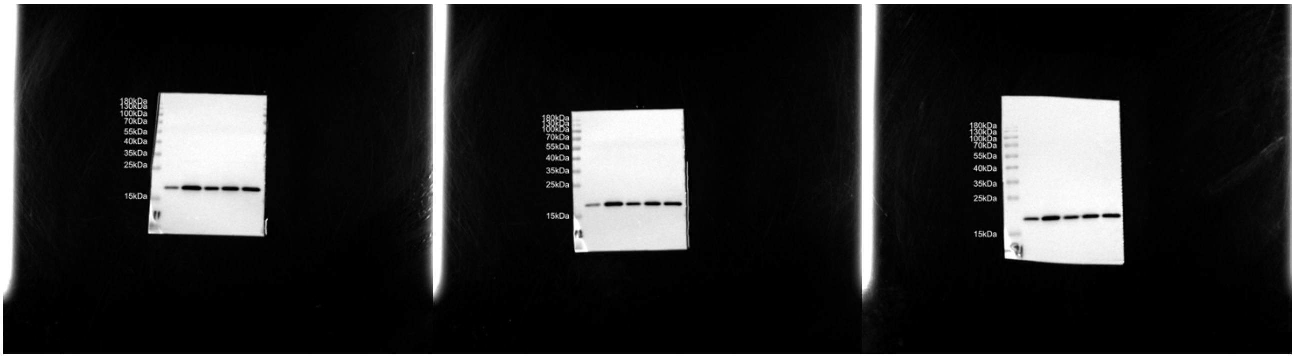

C-CAS3

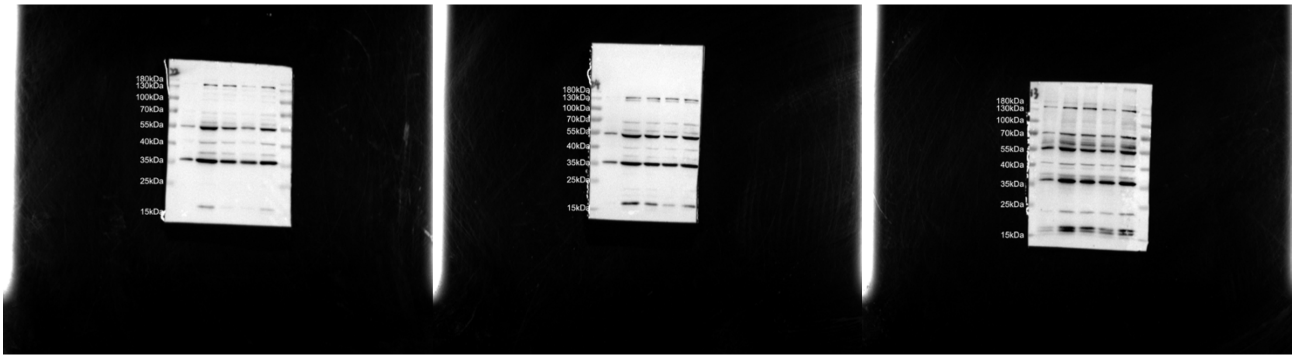

N-GSDMD

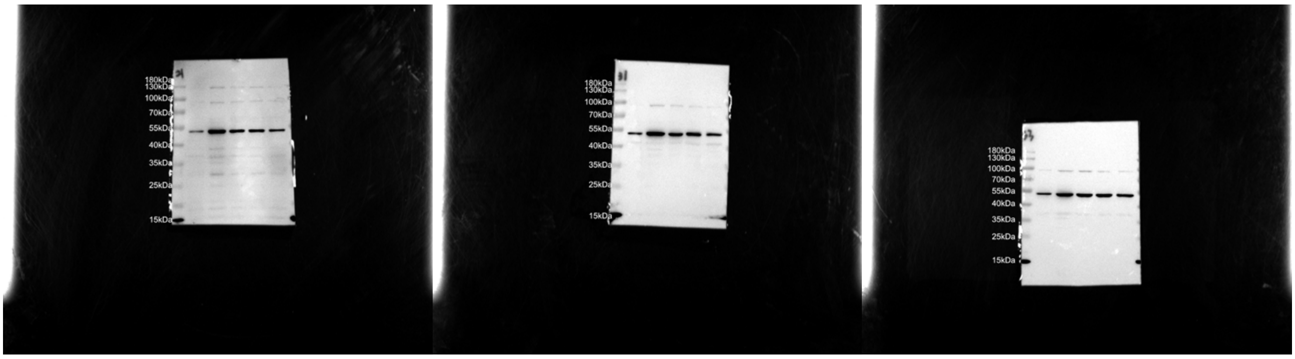

P-MLKL

Figure6A

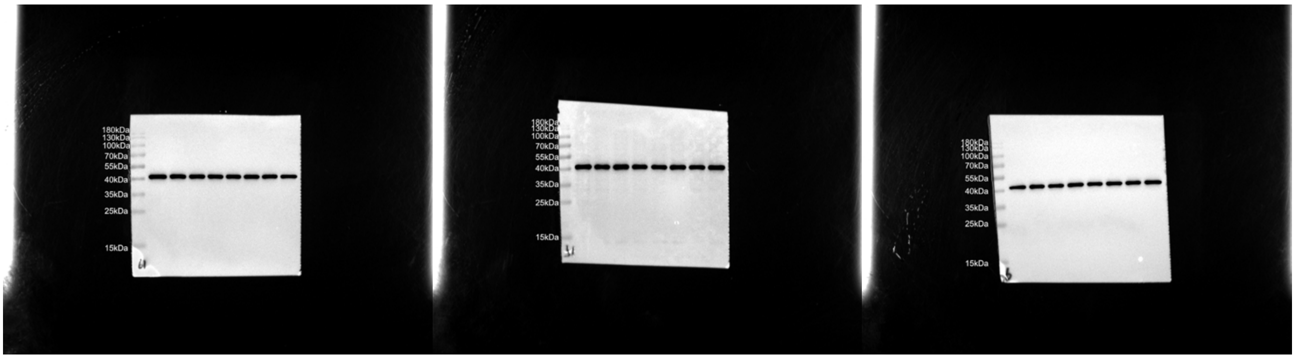

ACTIN

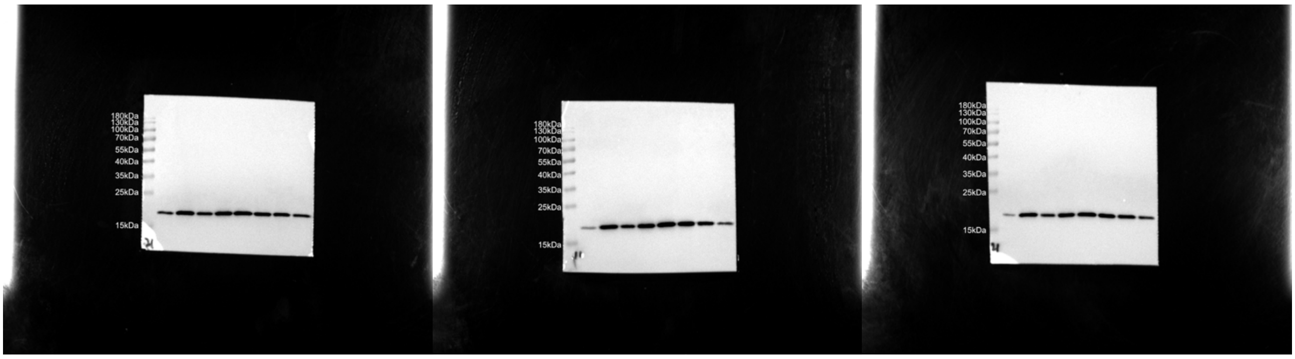

C-CAS3

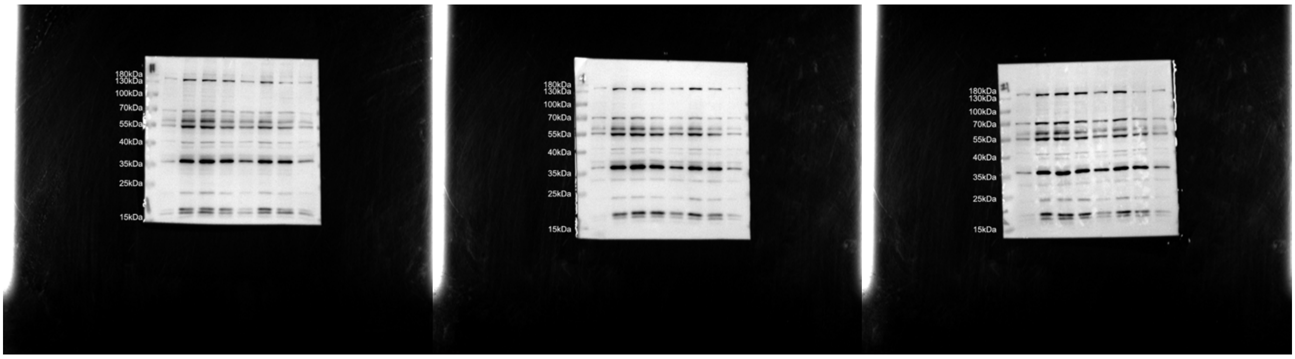

N-GSDMD

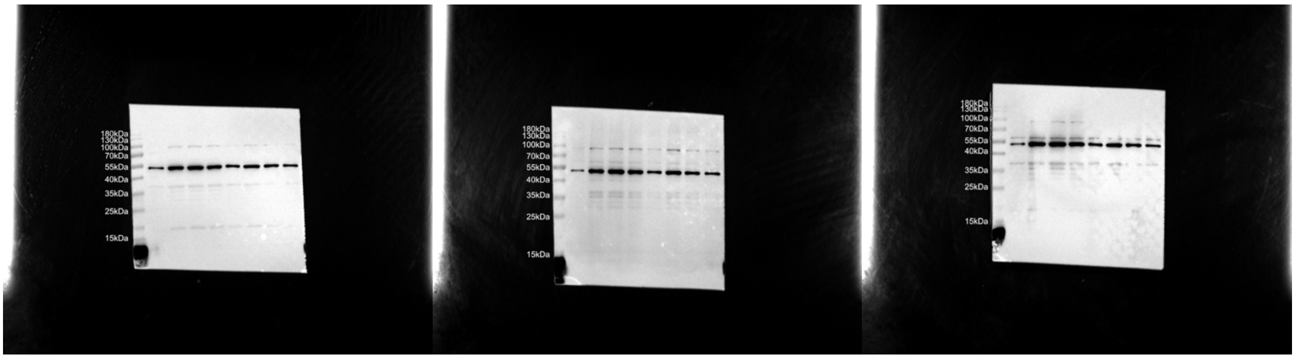

P-MLKL

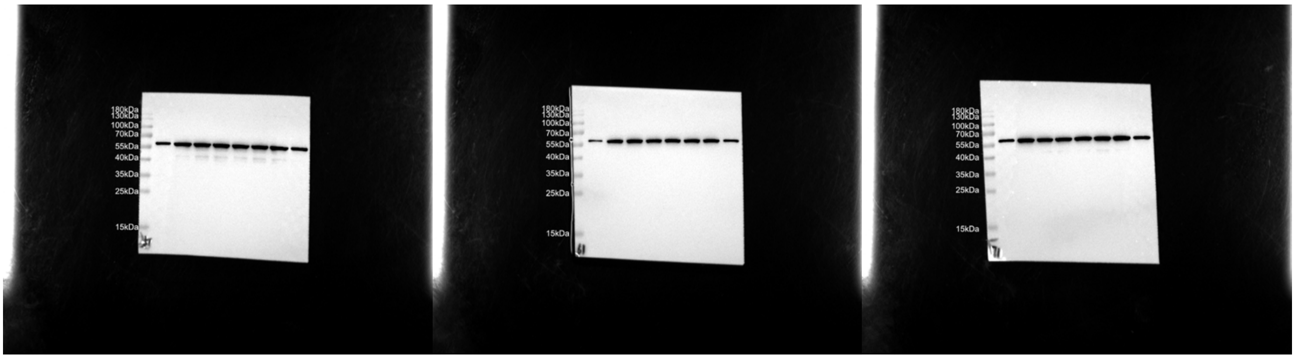

ZBP1

Figure7A

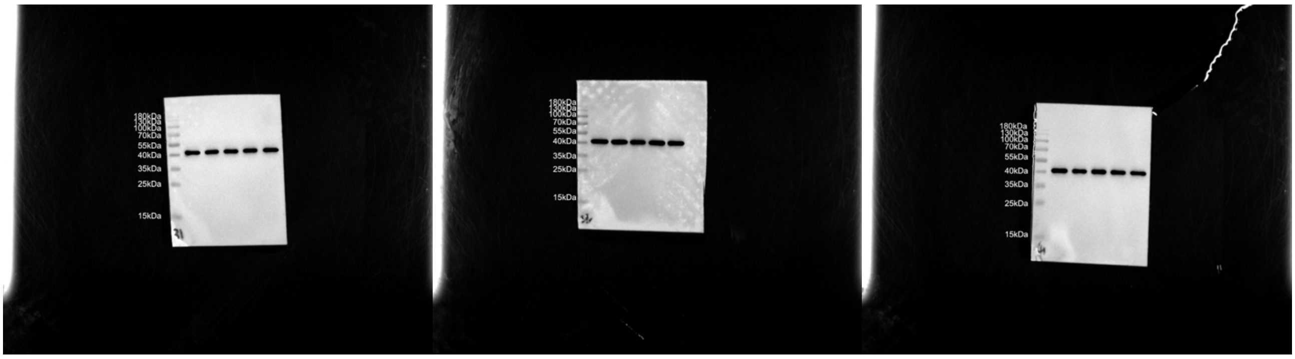

ACTIN.

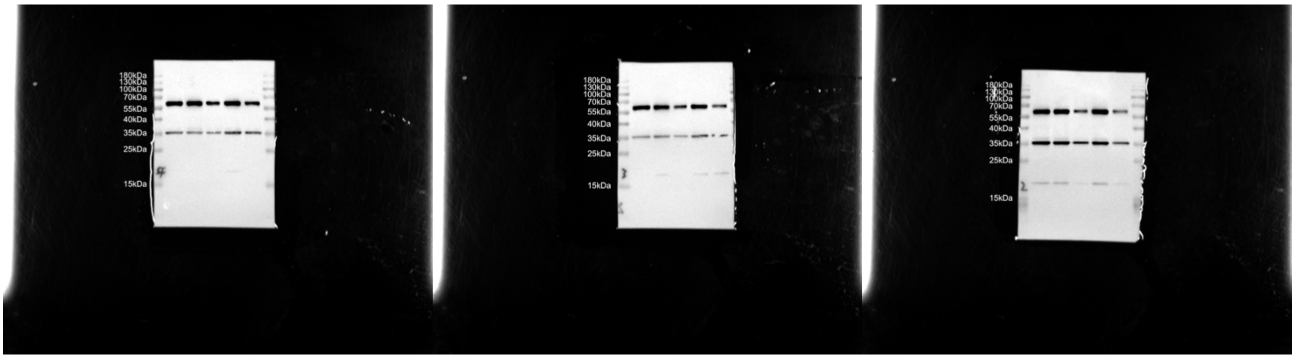

BIRC3.

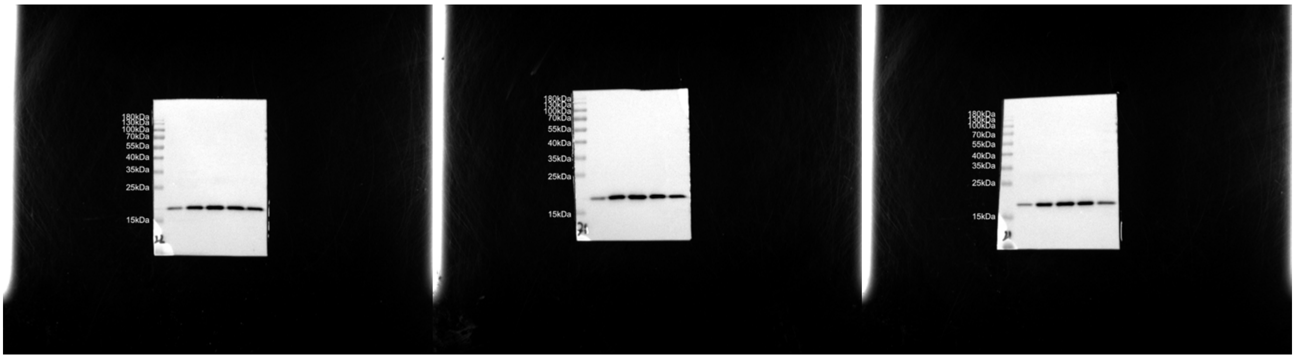

C-CAS3.

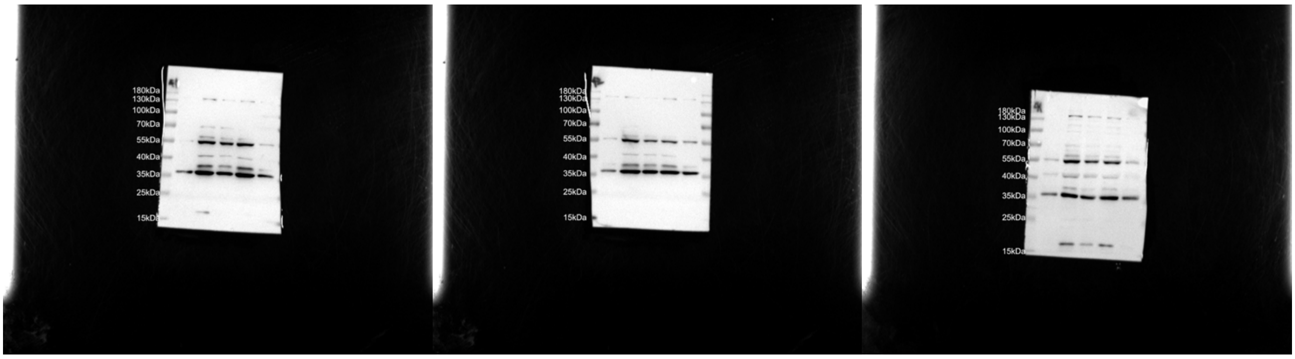

GSDMD-N.

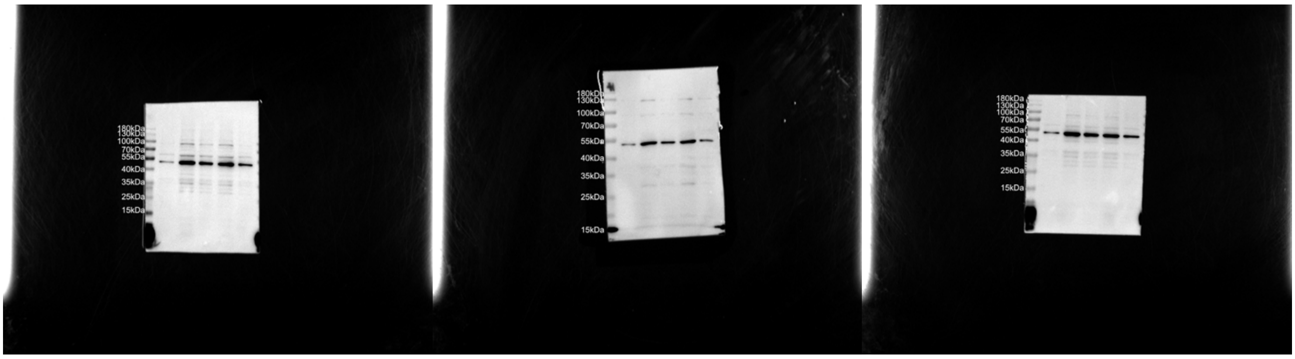

P-MLKL.

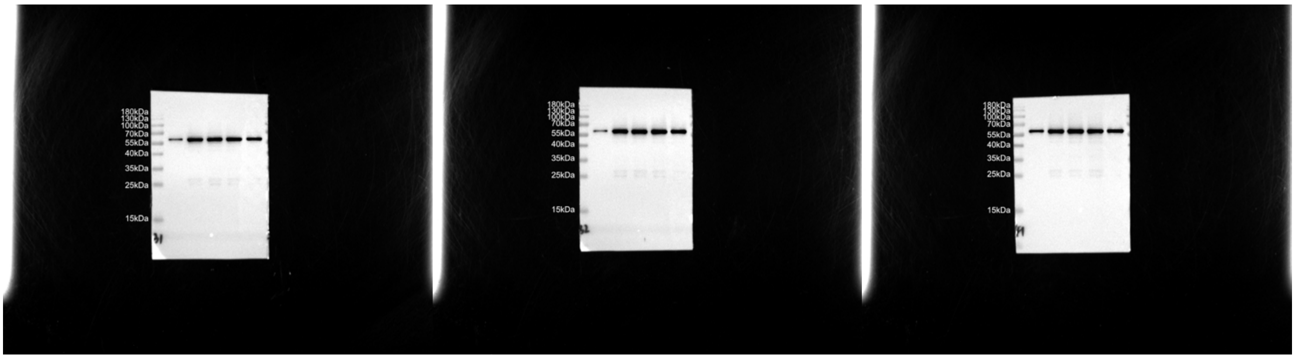

ZBP1.
